# Rap1b-loss increases neutrophil lactate dehydrogenase activity to enhance neutrophil migration and acute inflammation *in vivo*

**DOI:** 10.3389/fimmu.2022.1061544

**Published:** 2022-11-25

**Authors:** Chanchal Sur Chowdhury, Elizabeth Wareham, Juying Xu, Sachin Kumar, Matthew Kofron, Sribalaji Lakshmikanthan, Magdalena Chrzanowska, Marie-Dominique Filippi

**Affiliations:** ^1^ Division of Experimental Hematology and Cancer Biology, Cincinnati Children’s Research Foundation, Cincinnati, OH, United States; ^2^ University of Cincinnati College of Medicine, Cincinnati, OH, United States; ^3^ Division of Developmental Biology, Cincinnati Children’s Research Foundation, Cincinnati, OH, United States; ^4^ Versiti Blood Research Institute, Milwaukee, WI, United States; ^5^ Department of Pharmacology and Toxicology, Medical College of Wisconsin, Milwaukee, WI, United States; ^6^ Cardiovascular Center, Medical College of Wisconsin, Milwaukee, WI, United States

**Keywords:** neutrophils, inflammation, Ldha, migration, vascular leakage

## Abstract

**Introduction:**

Neutrophils are critical for host immune defense; yet, aberrant neutrophil tissue infiltration triggers tissue damage. Neutrophils are heterogeneous functionally, and adopt ‘normal’ or ‘pathogenic’ effector function responses. Understanding neutrophil heterogeneity could provide specificity in targeting inflammation. We previously identified a signaling pathway that suppresses neutrophilmediated inflammation via integrin-mediated Rap1b signaling pathway.

**Methods:**

Here, we used Rap1-deficient neutrophils and proteomics to identify pathways that specifically control pathogenic neutrophil effector function.

**Results:**

We show neutrophil acidity is normally prevented by Rap1b during normal immune response with loss of Rap1b resulting in increased neutrophil acidity via enhanced Ldha activity and abnormal neutrophil behavior. Acidity drives the formation of abnormal invasive-like protrusions in neutrophils, causing a shift to transcellular migration through endothelial cells. Acidity increases neutrophil extracellular matrix degradation activity and increases vascular leakage in vivo. Pathogenic inflammatory condition of ischemia/reperfusion injury is associated with increased neutrophil transcellular migration and vascular leakage. Reducing acidity with lactate dehydrogenase inhibition in vivo limits tissue infiltration of pathogenic neutrophils but less so of normal neutrophils, and reduces vascular leakage.

**Discussion:**

Acidic milieu renders neutrophils more dependent on Ldha activity such that their effector functions are more readily inhibited by small molecule inhibitor of Ldha activity, which offers a therapeutic window for antilactate dehydrogenase treatment in specific targeting of pathogenic neutrophils *in vivo*.

## Highlights

1. Ldha-induced acidity is key regulator of neutrophil migration.
2. Ldha activity increases neutrophil-mediated inflammation *in vivo*.
3. Inhibition of Ldha activity reduces vascular leakage and I/R injury.

## Introduction

Aberrant neutrophil infiltration into tissue is associated with tissue damage, sepsis, and numerous cardiovascular diseases such as ischemia-reperfusion (I/R) injury, atherosclerosis, and small vessel vasculitis (1). Neutrophils have long been thought to be a homogenous entity with pre-programmed effector functions. Yet, it is now recognized that neutrophil functions are plastic and heterogeneous and can adapt to specific inflammatory environment. Depending on the tissue and intensity of inflammation, subsets of neutrophils can exhibit distinct lifespans, synthesize various amount of cytokines, or adopt different migratory behaviors such that neutrophils are now viewed as ‘normal’ or ‘pathogenic/inflammatory’ cells (2–4). Understanding the molecular mechanisms driving neutrophil functional plasticity will help in designing new treatment options aimed at targeting specifically inflammatory neutrophils.

Neutrophil migrate across the blood vessel *via* a well-established multiple step cascade, known as the extravasation cascade. After an initial capture and rolling of floating neutrophils onto endothelial cells (EC), neutrophils adhere and crawl on the EC surface lumen to find a permissive site for transmigration across the endothelial barrier. Neutrophils can cross the EC barrier *via* two distinct routes, either between two endothelial cells - i.e., para-cellular route – or directly through them- i.e., trans-cellular route (5). The route of transmigration depends on the vascular bed of the tissue, as well as the intensity of tissue inflammation (5) and is favored with increased adhesiveness of EC, tightness of EC junctions and increased chemokine concentration (6). Neutrophils can also migrate away from the sites of inflammation, contributing to resolution or dissemination of inflammation (7). Our understanding of neutrophil migration plasticity is currently very limited.

We previously identified a signaling pathway that suppresses neutrophil inflammatory responses. The Ras proximity 1b (Rap1b) (8, 9) is recruited to neutrophil uropod and limits neutrophil hyperactivity. Neutrophils deficient in Rap1b have enhanced adhesive properties and integrin-mediated ROS production. They extend abnormal invasive protrusions and exploit the transcellular route of migration under normal inflammatory conditions, which results in increased neutrophil infiltration into tissue and tissue damage (10). Here, using global proteomics profiling of neutrophil invasive protrusions, we identified lactate-mediated milieu acidity as being sufficient to drive neutrophil inflammatory phenotype. Acidic neutrophils can upregulate transcellular migration, increase neutrophil invasive actin protrusions in a manner dependent on Akt/HIF1a/Lactate dehydrogenase (Ldha) signaling *in vitro*, and enhance vascular injury *in vivo*. Acidic neutrophils are highly dependent on Ldha activity and are inhibited by low concentrations of Ldha inhibitor contrary to normal neutrophils. Pathogenic neutrophil phenotype is observed in inflammatory disease condition of I/R injury *in vivo*. Together, our study suggests that lactate-mediated acidity drives pro-inflammatory neutrophils that may be specifically targeted with small molecule inhibitors to reduce pathological inflammatory conditions without blocking completely innate immune functions.

## Methods

### Chemicals and reagents

*See online* **Supplementary Table S4**.

### Mice strains

The Rap1b-null mice (Rap1b^-/-^; and Tie2-Cre^+/0^ Rap1b^-/-^) were described previously (11, 12). All experimental procedures and animal protocols were approved by the Cincinnati Children’s and Medical College of Wisconsin’s Institutional Animal Care and Use Committees in accordance with AAALAC accreditation standards.

### Immunocytochemistry

All samples were fixed with 2% paraformaldehyde (PFA) for 20 min at room temperature (RT). After washing with PBS, cells were permeabilized with 0.1% Triton™ X-100 (Sigma) for 5 min. Non-specific sites were blocked with 5% BSA for 1 h followed by incubation with primary and secondary antibodies. For 2D migration assay 0.1% Saponin in 2% BSA was used for blocking and immunostaining. *See supplemental image acquisition and processing.*

### Neutrophil Isolation

Bone marrow (BM) neutrophil isolation was performed as using Histopaque gradient centrifugation as described previously (13).

### Neutrophil polarization assay

Neutrophils were stimulated with fMLP in HBSS containing 0.1% BSA on chambered glass slides for 10-15 min at 37°C. All samples were fixed with 2% PFA at for 20 min and permeabilized with 0.1% Triton X-100 before immunostaining.

### Transwell protrusion assay and quantification

BM neutrophils in HBSS buffer were added over 24 well, 1µm, transparent PET Transwell^®^ inserts and fMLP chemotaxis was applied from lower chamber for 15 min and 60 min time points. When indicated, neutrophils were pretreated with indicated inhibitor/activator or vehicle control for 45 min at 37°C. Post PFA fixation and washing membrane was cut out of the transwell and immunostained. Using Surface-rendering software Imaris, immunofluorescent signal of the protrusions were converted into 3D models and volume quantification was performed using computational algorithms of Imaris software. Hoechst-positive nuclei were counted to normalize data between groups.

### Mass spectrometry of invadosome

Transwell protrusion assay was performed onto 6 well, 1µm chamber (see above). Invadosomes were extracted as published (14, 15). Using a PBS-dipped cotton bud, neutrophil cell bodies from top of the filter were removed by swiping over the membrane. Invadosome protein fractions left in the pores of the chamber were lysed using RP1 buffer and purified by NucleoSpin^®^ RNA/Protein kit. Isolated protein fractions were analyzed by MS **using mass spectrometry facility of Ohio State University.** *See supplemental methods for bioinformatics.*

### Chemotaxis assay

Chemotactic migration was recorded in transwell chambers as described previously (16). See supplemental methods for experimental details.

### In-vitro transendothelial migration (2D migration) assay

Neutrophil migration over LPS activated bEND.3 murine brain endothelial cells was performed as previously detailed (10). *See supplemental methods for experimental details.*

### LPS-induced lung inflammation

Mice were challenged with 1.25 mg/kg LPS from Escherichia coli O111:B4 by intratracheal instillation after ketamine and xylazine anesthesia and BALF was collected as described previously. When indicated, mice were given intraperitoneal injection of DMSO or 3µg of FX11 (0.15mg/kg body weight) dissolved in PBS, 1 h before LPS instillation.

### Lactate quantification

Supernatants were collected after 15 min of stimulation of neutrophils with fMLP or vehicle control (DMSO). Lactate quantification assay and analysis was performed using Biovision Lactate Colorimetric/Fluorometric Assay Kit as per manufacturer instructions.

### Intracellular acidification measurement

WT or Rap1b^-/-^ neutrophils were added to glass bottom chambered slides in HBSS and allowed to settle to the bottom of the plate for 45 min at 37°C. After incubation, cells were treated with solution containing pHrodo™ Red and fMLP for 10-15 min at 37°C and imaged under live microscope. In addition, neutrophils were resuspended in HBSS media at 1X10^6^ cells/ml and incubated with 100nM BCECF-AM for 10 min at 37°C in the presence or absence of FMLP stimulation. GFP fluorescence was analyzed by flow cytometry.

### Extracellular milieu acidification conditions

HCI solution was added to the HBSS + 10% FCS medium, which contained bicarbonate, to adjust the pH value of the medium.

### *In vivo* transmigration and vascular leakage model

Ear of mice were stimulated and blood vessels were marked by intradermal injection of fMLP and Pecam-1 in PBS. Neutrophil migration and vascular leakage were detected by retro-orbital injection of fluorescent dye conjugated Ly6G and Dextran. *See supplemental methods for experimental details.*

### Competitive *in vivo* migration assay

WT and Rap1b^-/-^ neutrophils were distinctly labeled (WT neutrophils with cell tracker red and Rap1b^-/-^ neutrophils with cell-tracker Red plus Hoechst) and adoptively transferred into recipient albino mice in 1:1 ratio. Alexa 488 conjugated Pecam-1 antibody was used to mark vascular endothelial junctions, and neutrophil migration was induced with intradermal fMLP injection in the ear. *See supplemental methods for experimental details.*

### Ear ischemia reperfusion injury

I/R injury of the ear was performed as described previously (17). *See supplemental methods for experimental details.*

### Statistical analysis

Group comparisons was performed using the Student t test. Results were summarized in terms of least squared adjusted means and standard errors, using GraphPad Prism. P values are labeled as *P <.05, **P <.01, ***P <.001, and ****P <.0001.

## Results

### Increased intracellular acidity is associated with increased neutrophil protrusions

**Figure 1:**
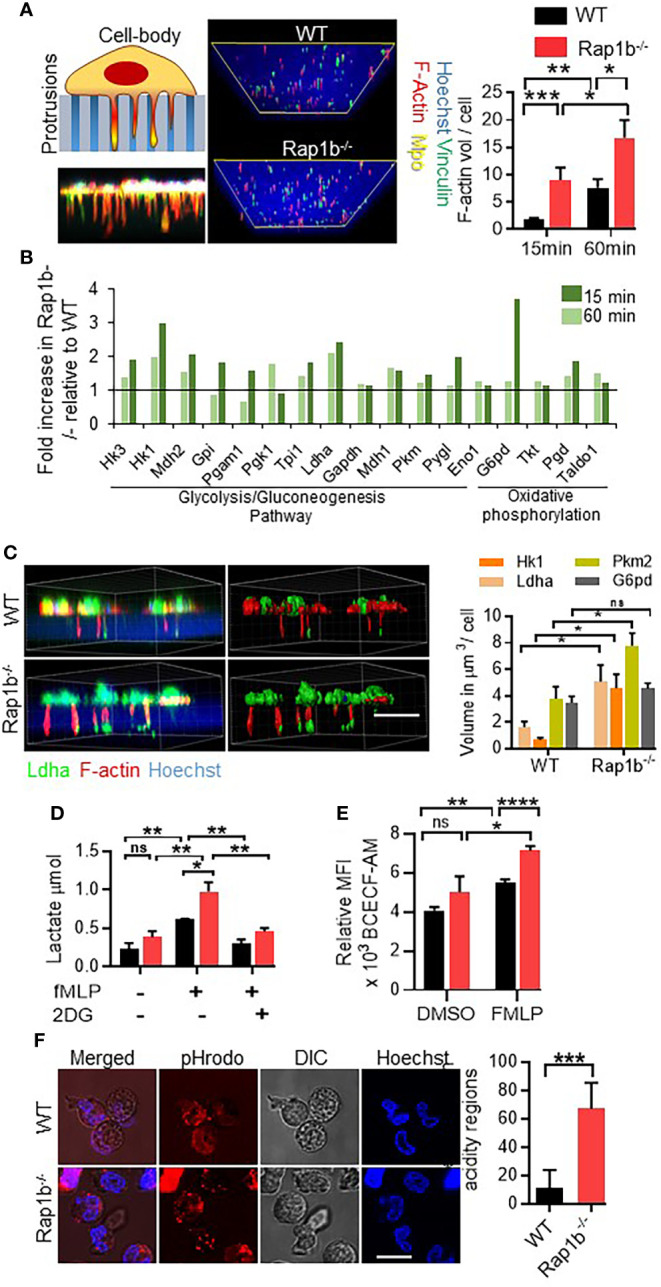
Rap1b deficiency enhances Ldha activity in neutrophils. **(A)**, Analysis of neutrophil invasive protrusions after fMLP chemotaxis, over 1µm transwell filter. Schematic diagram and representative confocal z-stack image showing trapped neutrophil protrusions in the porous membrane of transwell filter (left panel). Representative Z-stack sections from protrusion fractions of Rap1b^-/-^ and WT neutrophils were rebuilt using Imaris software (right panel). Images are the view of the protrusions from the bottom of the filter. Bar graph represents the volume of F-actin positive protrusions per 100 WT and Rap1b^-/-^ neutrophils, at 15min and 60min time points. (n=8 independent experiments, average of 60cells each group each condition analyzed). **(B)**, Mass spectrometric analysis showing percent change in metabolic enzymes in the protrusive fractions of Rap1b^-/-^ and WT neutrophils at 15 min and 60 min time points (n=1, pooled neutrophils from 2 mice each condition). **(C)**, Immunostaining analysis of fixed WT and Rap1b^-/-^ neutrophils after 15 min of fMLP chemotaxis on 1µm transwell filters. Representative fluorescent image showing localization of Ldha (green) along with F-actin (red) positive protrusions (Left). Image rebuilt using Imaris on which volume quantification was performed (Right). Bar graph showing relative volume of Ldha, G6pd, Pkm2 and Hk1 positive staining in the protrusion fraction. (n=3 independent experiments. Average of minimum 100 cells/group/experiment). **(D)**, Quantification of Lactate release by adherent neutrophils after fMLP stimulation (n=3 independent experiment). **(E)**, Isolated neutrophils were incubated with BCECF-AM for 10 min at 37°C in the presence or absence of FMLP and pHi was measured using flow cytometry. (n=3 mice per group per condition. 3 independent experiments) **(F)**, Representative images showing region of intracellular acidification by pH sensitive dye, pHrodo, in live WT and Rap1b^-/-^ neutrophils after 15min of fMLP stimulation. Bar graph represents quantification of percentage positive cells per field (n=6 fields under 60x objective per condition; 2 independent experiments). Means ± SEM. For all panels: *P < 0.05; **P < 0.001; ***P < 0.0004; ****P < 0.0001; NS, not significant using unpaired Student’s t test. Scale bar; (A,C,D,G)=10µm.

We previously reported that Rap1b^-/-^ neutrophils extend long protrusions with enhanced metalloproteinase activity and adopt a transcellular migration behavior (10). To further understand mechanisms that drive this phenotype, we used global proteomics profiling of neutrophil protrusions (*see methods*) extended by WT and Rap1b^-/-^ neutrophils *in vitro* (**Figure 1A**). We first examined these structures using immunofluorescence and showed that they were made of F-actin and vinculin. They contained the membrane and cytoskeletal markers CD44 and Arp-3, respectively, but were devoid of the cytoplasmic marker Myosin 2b and the nuclear stain Hoechst (***Online*** **Figure S1A**). Volume quantification method of Imaris software, confirmed Rap1b^-/-^ neutrophils extended more F-actin-rich protrusions than WT neutrophils (**Figure 1A**). We then isolated these protrusions for proteomics analysis (***Online*** **Figure S1B**). Protrusions were harvested at two different time points of stimulation, 15min (early) and 60min (late) to understand the kinetics changes. To streamline the analysis, we focused on the consensus list of proteins with minimum cutoff of 5 peptides in each group. To identify pathways that sustain the formation of protrusions and are physiologically relevant, we focused on the 190 proteins that were present in the protrusions of all 4 groups (***Online*** **Figure S1C** ***, Online*** **Table S1**). Gene Ontology analysis indicated that these proteins belonged to ‘focal adhesion’ and ‘actin cytoskeleton’ categories, as expected, as well as proteins responsible for ‘protein binding’, especially actin filament binding proteins (***Online*** **Figure S1D** ***, Online*** **Tables S2-S3**). To analyze differences in the protein composition of Rap1b^-/-^ and WT protrusions, the cutoff was set at 1.2 fold change between the groups. Pathway analysis suggested Rap1b^-/-^ protrusions had higher content of proteins belonging to ‘innate immune signaling’, ‘RhoGTPase effector signaling’, and interestingly ‘carbon/glucose metabolism’ (***Online*** **Figure S1E**). Metabolic pathways such as glycolysis and gluconeogenesis were the most enriched pathways in Rap1b^-/-^ protrusions at 15 min with higher expression of glycolysis/gluconeogenesis enzymes including phosphoglycerate kinase 1 (Pgk1), lactate dehydrogenase (Ldha), pyruvate kinase (Pkm2) and hexokinase 1 and 3 (Hk1/3) (**Figure 1B**). Ldha expression was 2-fold higher in Rap1b^-/-^ protrusions both at 15 min and 60 min of stimulation. Immunofluorescence analysis of protrusions formed in 1 μm transwell filters within 15 min of fMLP stimulation, confirmed higher levels of Ldha, Pkm2 and Hk1 in Rap1b^-/-^ protrusions compared to WT (**Figure 1C**). Specifically, Ldha was enriched at the tip of the protrusions. Hence, protrusions extended by Rap1b-deficient neutrophils are enriched for metabolic enzymes.

Ldha catalyzes the conversion of pyruvate to lactate, which in turn increases intracellular and extracellular acidity. To assess whether enhanced Ldha expression was associated with enhanced activity, amount of lactate released after fMLP stimulation was examined. Compared to WT neutrophils, Rap1b^-/-^ neutrophils released higher levels of lactate, and this is dependent on glucose uptake since inhibition of glycolysis by 2-Deoxy-D-glucose (2-DG) completely inhibited lactate release (**Figure 1D**). Moreover, fMLP stimulation rapidly increased intracellular acidity in Rap1b^-/-^ neutrophils as measured by flow cytometry using the fluorescent probe BCECF-AM (**Figure 1E**). Additionally, using immunostaining with pH sensitive dye pHrodo on live cells, Rap1b^-/-^ neutrophils displayed intracellular regions with high acidity levels compared to WT neutrophils, in response to fMLP stimulation (**Figure 1F** *and Online* **Figure S2**), consistent with increased lactate production. Hence, Rap1b-deficiency increases neutrophil acidity.

### Pharmacological inhibition of Ldha activity inhibits both *in vitro* and *in vivo* migration of Rap1b^-/-^ neutrophils

**Figure 2:**
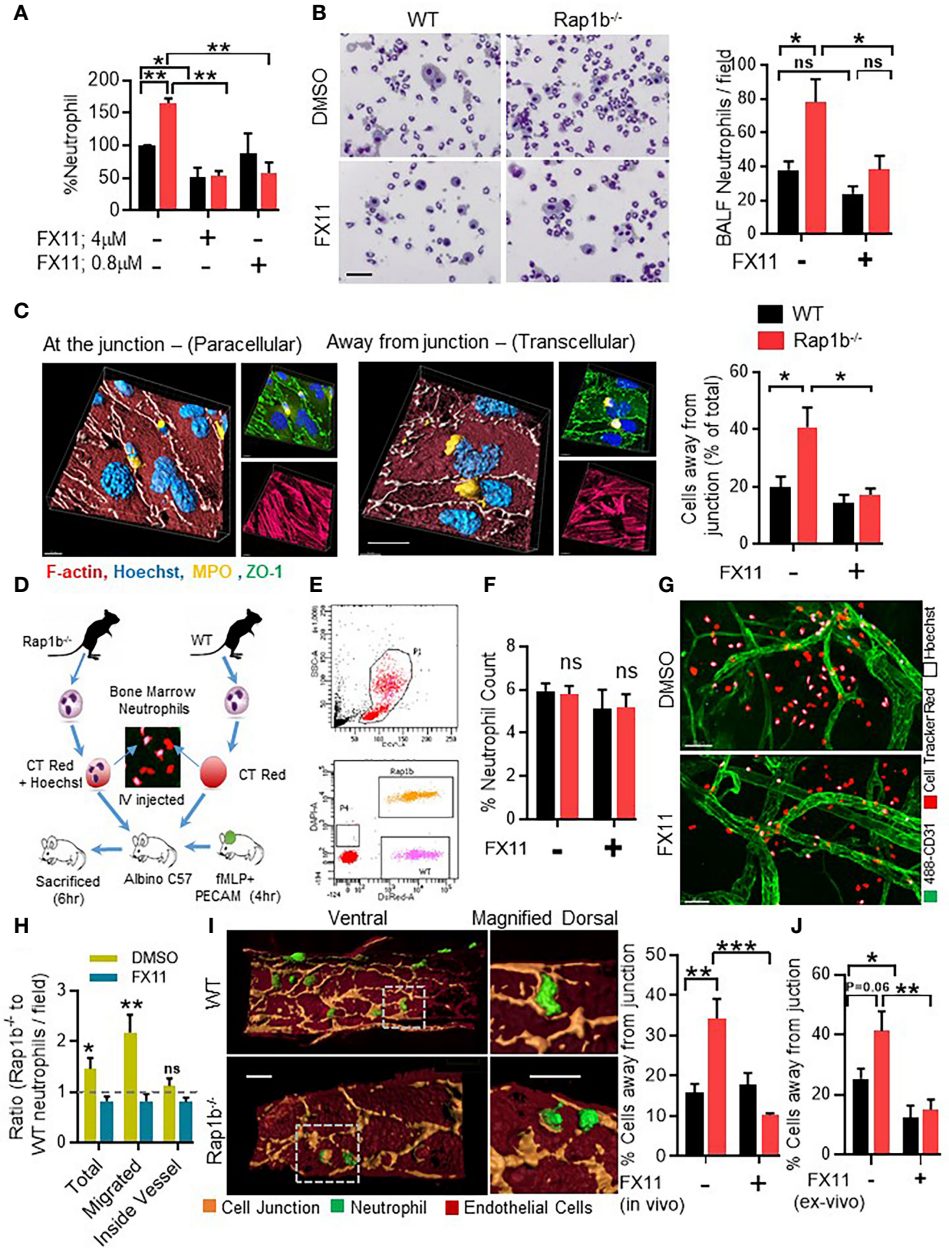
Ldha inhibition reduces neutrophil transcellular migration *in vitro* and *in vivo*. **(A)**, Migration of whole neutrophil body though 3µm transwell filter pores after fMLP chemotaxis. Percent neutrophil count, relative to WT control group that migrated through fibrinogen coated 3µm transwell filters after 3 h fMLP chemotaxis in the presence or absence of Ldha-I at indicated concentrations. (n=3 mice for each plot, 3 independent experiments). **(B)**, BALF analysis of mice reconstituted with WT or Rap1b^-/-^ hematopoietic cells that were challenged with interstitial saline or LPS (1.25 mg/kg) for 4 h, in the presence of either FX11 or vehicle control (DMSO). Representative area showing relative number of cells under 20 x objective, in equal volume of BALF samples taken for cytospin. Bar graph (left) shows BALF neutrophil count per field of cytospin samples under 20x objective. (n=6 mice per group; average of 10 fields/sample/experiment. 3 independent experiments). **(C)**, *In vitro* neutrophil transendothelial migration over LPS activated Bend3 monolayer. Representative 3D images (Left) built on fluorescent Z-stack images (right) showing neutrophil location after adhesion and locomotion onto LPS-activated bEND.3 (2D Migration assay), stained with ZO-1 (green), MPO (yellow) and Hoechst (blue). Bar graph represents percentage of cells located away from junction after pretreatment with DMSO or Ldha-I (0.1µM). (n=5 independent experiment; average of 10 fields under 60x objective/condition/experiment analyzed). **(D-H)**, *In vivo* competitive migration assay where fluorescent tagged WT and Rap1b^-/-^ neutrophils were adoptively transferred to recipient albino mice having fMLP stimulated ear vasculature in the presence of FX11 or DMSO. Schematic illustration of *in vivo* competitive migration assay **(D)**. Scattered plot of peripheral blood cells isolated after 4 h of adoptive transfer, showing presence of fluorescent stained Rap1b^-/-^ (orange) and WT (pink) neutrophil populations **(E)**. **(F)**, Bar graph represents percent neutrophil count in the peripheral blood (n=6 mice/plot; 3 Independent experiments). **(G)**, Inflamed mouse ear vasculature stained with Pecam-1(green), Rap1b^-/-^ neutrophils (CellTracker red+ Hoechst) and WT neutrophils (CellTracker red). White arrows indicate regions of high acidity. **(H)**, Bar graph represents ratio of Rap1b^-/-^ and WT neutrophils, that migrated out of vessels or stayed within the blood vessels (n=8 fields/condition/experiment; 3 Independent experiments). **(I)**, Representative 4D images showing *in vivo* transmigration, built over immunofluorescent images of ear vessel stained with anti-Pecam-1 (pseudo colored to light brown), endothelial cells body (dark brown) and neutrophils (green) using surface module of Imaris software. Bar graph showing percent of neutrophils away from endothelial junction (N=4 independent experiments; 5 areas/group/experiment). **(J)**, Bar graph showing percentage neutrophils migrating away from vessel junction after ex vivo FX11 (0.1µM) or DMSO pretreated and cell tracker dye labeled neutrophil were adoptively transferred into mice with fMLP stimulated ear vasculature for 2 h. (n=6 fields per data set. 2 independent experiments). Mean ± SEM; *P < 0.05; **P < 0.001; ***P < 0.0004; NS, not significant using unpaired Student’s t test. Scale bar; **(B, C, I)**=20µm, **(G)**=50µm.

Ldha activity is necessary for normal neutrophil functions. Therefore, to assess the functional importance of enhanced Ldha activity in neutrophil migration, we used a highly specific, reversible, and competitive pharmacological inhibitor of Ldha activity, FX11. This approach allows us to reduce Ldha activity without completely abrogate it, and to use varying concentrations of the inhibitor in order to examine sensitivity of neutrophil responses, which cannot be done with a genetic approach. Pretreatment with FX11 inhibited lactate production in both WT and Rap1b^-/-^ at ED50 concentration (4µM) whereas at lower concentration (0.08µM), lactate production was inhibited only in Rap1b^-/-^ neutrophils (***Online*** **Figure S3A**). In the transwell assay *in vitro*, Rap1b^-/-^ neutrophil migration was increased compared to wild-type (WT) in response to fMLP (**Figure 2A**). In the presence of normal concentration of FX11 (4 µM), both WT and Rap1b^-/-^ neutrophil migration were inhibited. However, a lower concentration of FX11 (0.08µM) was sufficient to inhibit Rap1b^-/-^ neutrophil migration without affecting WT neutrophil migration. Effect of Ldha inhibition on neutrophil migration was also evaluated *in vivo* in a LPS-driven acute lung inflammation model (10). WT mice were reconstituted with WT or Rap1b^-/-^ bone marrow (BM) cells to investigate neutrophil cell-intrinsic effects. Rap1b^-/-^ reconstituted mice had increased neutrophil infiltration into inflamed lungs, compared to WT reconstituted mice, in the bronchoalveolar lavage (BAL) fluid (**Figure 2B**). Interestingly, this was significantly inhibited with LdhaI treatment *in vivo*, 1 hour prior to LPS challenge, suggesting that Rap1b loss-mediated acidity enhances neutrophil migration *in vivo*.

We next examined the role of Ldha activity on the route of transmigration using 2D migration assay (see methods). Under these conditions, neutrophils adhere and interact with EC, at the junction or away from it within the EC body, and transmigrate. In this assay, a transmigratory cup characterized by a ring of ICAM is formed when the cells interact with EC and migrate at or away from the EC junction (***Online*** **Figure S3B**). Neutrophil/EC interactions and ICAM cup formation located away from the EC junction is an indication of transcellular migration (10, 18, 19). Using these parameters, WT neutrophils were mostly at the EC junctions. In contrast, higher frequency of Rap1b-/- neutrophils were located away from the EC junction *(* **Figure 2C** *and* **Figures S3B, C**). Pretreatment with FX11 significantly reduced the number of neutrophils away from EC junction, in WT and Rap1b^-/-^ neutrophils. Similar results were obtained after pretreatment with another potent, cell permeable and reversible pharmacological inhibitor of Ldha, GSK2837808A (LdhaI-II) (***Online*** **Figure S4A**).

To study this *in vivo*, we designed a competitive migration assay (**Figure 2D**, *see methods*) to quantify the relative migration of WT and Rap1b^-/-^ neutrophils in the same vascular microenvironment. Two hours after adoptive transfer, the percent of WT (labeled with dsRED) and Rap1b^-/-^ neutrophils (labeled with DAPI and dsRED) in the peripheral blood of recipient albino mouse was equal (**Figures 2E, F**). In contrast, after fMLP challenge, the overall number of Rap1b^-/-^ neutrophils [DAPI and dsRED cells] present at the site of inflammation was higher than WT cells [dsRED cells] (**Figure 2G**). The distribution of neutrophils, i.e. inside or outside of the blood vessel in the interstitial tissue, per inflammatory sites, was then examined. The number of WT and Rap1b^-/-^ neutrophils estimated to be inside blood vessels were similar. In contrast, higher numbers of Rap1b^-/-^ neutrophils were found in the tissue as indicated by increased ratio of Rap1b^-/-^ to WT neutrophils in tissue relative to blood (**Figure 2H**). Interestingly, using imaris software analysis, we can capture neutrophils that are in the act of migration and form a PECAM-ring in the EC body (shown in **Figure 2I**). We noted the proportion of neutrophils with a PECAM ring and positioned in the EC bodies was higher in Rap1b-/- than in WT neutrophil migration *in vivo*. (**Figure 2I**) We interpret neutrophils surrounded by a ring of PECAM in the EC body to have distinct neutrophil/EC interactions and use transcellular migration. Finally, neutrophils were treated with FX11 ex vivo and then adoptively transferred into mice. Neutrophil migration was evaluated 2 hours after fMLP challenge. In this assay, the number of Rap1b-/- neutrophils migrating away from EC junction was also reduced (**Figure 2J**) suggesting cell-intrinsic effect of Ldha inhibition. Together these results suggest that increased Ldha activity modifies neutrophil/EC interactions, route of migration and enhances neutrophil tissue infiltration during inflammation.

### Inhibition of Ldha activity reduces inflammation-induced vascular leakage

**Figure 3:**
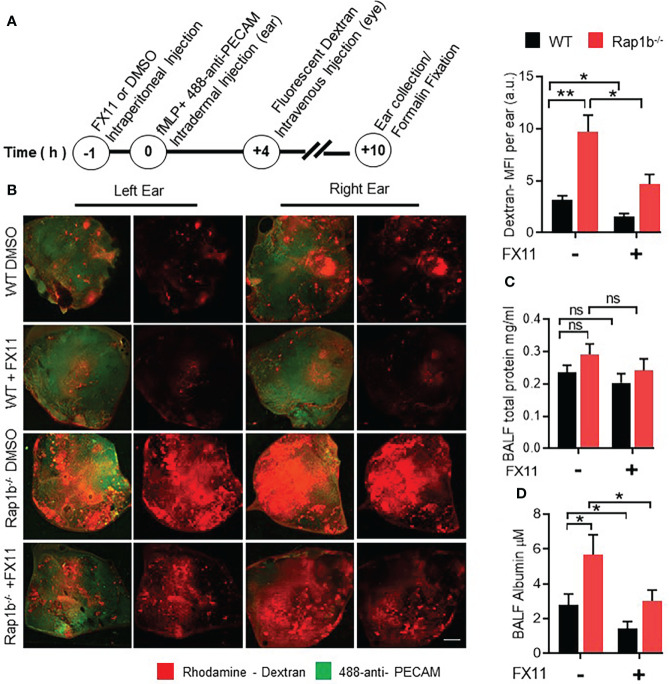
FX11 treatment decreases vascular leakage *in vivo*. **(A)**, Ear vascular leakage analysis in fMLP stimulated Rap1b^-/-^ and WT mice ear. Timeline depicting vascular leakage model in mouse ear. **(B)**, Representative fluorescent images of whole ear mount, showing amount of rhodamine dextran leakage following fMLP stimulation for 10 h in the presence or absence of Ldha-I. Bar graph showing quantification of total rhodamine dextran fluorescence per ear, after 10 h of fMLP stimulation in the ear (n=4 mice each group; 2 independent experiments). **(C, D)** BAL fluids were analyzed for vascular leakage after lungs were challenged with interstitial saline or LPS for 4 h in the presence or absence of Ldha-I in WT and Rap1b^-/-^ mice. Bar graphs show concentration of total protein **(C)** and total albumin **(D)**, in BALF after LPS challenge (n=6 mice/condition; 3 independent experiments). Mean ± SEM, *P < 0.05; **P < 0.001; NS, Not Significant, using unpaired Student’s t test. Scale Bar **(B)**=2mm.

To examine whether Rap1b^-/-^ neutrophil functions correlated with increased vascular permeability, Rap1b^-/-^ and WT reconstituted mice were injected with fluorescent dextran and neutrophil migration was induced in the ear vasculature with local intradermal injection of fMLP (**Figure 3A**). Rap1b^-/-^ reconstituted mice exhibited higher leakage of rhodamine-labeled dextran in the inflamed ear tissue than WT mice (**Figure 3B**). Intraperitoneal injection of FX11, 1hr prior to fMLP stimulation, reduced the extent of vascular leakage more prominently in Rap1b^-/-^ reconstituted mice than in WT mice. We also tested the effect of Ldha inhibition on vascular leakage in the LPS lung inflammation model (10, 20). No significant differences in total protein content in the BALF 4h after LPS stimulation were found between the genotypes (**Figure 3C**). In contrast, the levels of albumin were significantly elevated in Rap1b^-/-^ BALF compared to WT (**Figure 3D**). This increase was reduced to almost WT levels in BALF of Rap1b^-/-^ reconstituted mice that had been treated with FX-11 prior to LPS challenge. Hence, Rap1b loss increases lung inflammation and tissue leakage in a manner dependent on enhanced LDHA activity.

### F-actin protrusions and extracellular matrix degradation is regulated by Ldha activity

**Figure 4:**
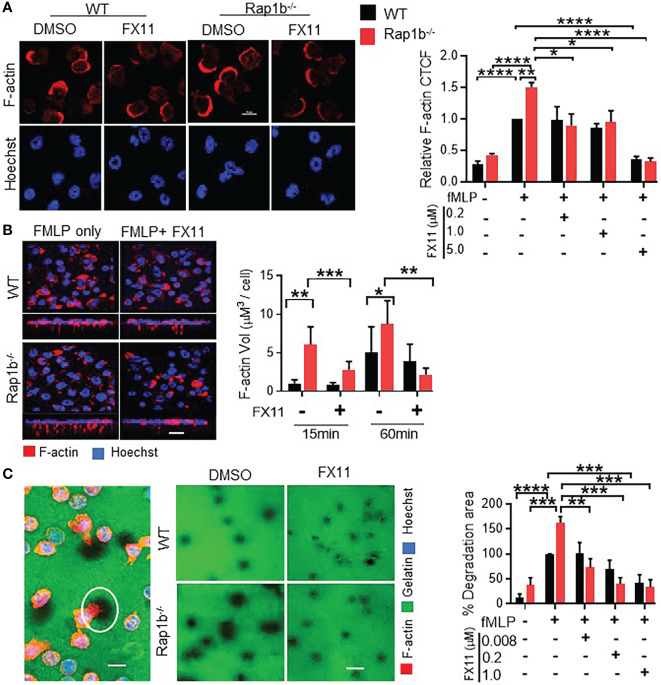
Formation of invasive protrusions and extracellular matrix degradation is dependent on neutrophil Ldha. **(A)**, Effect of Ldha inhibition on neutrophil F-actin polymerization. Representative images of WT and Rap1b^-/-^ neutrophils that were stimulated with fMLP in chambered glass slides for 10 min after pre-incubation with Ldha-I or vehicle control (DMSO) and stained with rhodamine/phalloidin (red) and Hoechst (blue). Bar graphs showing relative F-actin corrected total cell fluorescence (CTCF) normalized to WT vehicle control taken as 1 (n=4 independent experiments; average of 30 cells/per group/experiment). **(B)**, Analysis of protrusion formation on 1µm transwell filter at 15min in presence or absence of Ldha-I. Z-stack images of Rap1b^-/-^ and WT neutrophils showing formation of invasive protrusions as detected by staining of F-actin (red) and Hoechst (blue). Bar graphs represents volume of F-actin protrusions using Imaris software (n=3 independent experiments. Average of >200 cells/group/experiment). **(C)**, Extracellular matrix degradation by fMLP stimulated neutrophils. Representative image showing area of degradation as detected by dark areas (white circle) and staining of neutrophils with F-actin (red) and Hoechst (blue) (Right). Representative images showing gelatin degradation by WT and Rap1b^-/-^ neutrophils in the presence or absence of Ldha-I (Left). Bar graphs represent percent degradation area per field relative to WT DMSO group. (n=4 independent experiments; average of 150 cells/condition/experiment). (n=3 independent experiments; average of >10 fields under 60x objective/group/experiment). Mean ± SEM; *P < 0.05; **P < 0.001; ***P < 0.0004; ****P<0.0001; using unpaired Student’s t test. Scale Bar; **(A-C)**, = 10µm.

Mechanistically, we examined how acidity could modulate neutrophil migration. Two key aspects of neutrophil migration that appear to be specific of the transcellular pathway, ie increased actin protrusions associated with proteolytic activity, are elevated in Rap1b-/- neutrophils. In response to fMLP, WT neutrophils extend a single lamellipodia of F-actin at the cell front. In contrast, Rap1b^-/-^ neutrophils display increased F-actin polymerization in a form of a larger lamelipodia (10) (**Figure 4A**). Addition of FX-11 inhibited abnormal F-actin polymerization in Rap1b^-/-^ neutrophils. However, higher concentration of FX11 also inhibited F-actin polymerization of WT neutrophils. In 1 µm transwell, FX11 inhibited Rap1b^-/-^ neutrophil F-actin protrusions without altering those extended by WT neutrophils (**Figure 4B**). Pretreatment of WT and Rap1b^-/-^ neutrophils with another pharmacological Ldha inhibitor, GSK2837808A gave similar results (***Online*** **Figure S4B**).

Further we tested the effect of Ldha inhibition on the invasiveness of these protrusive structures. The local proteolytic activity of cell protrusions can be identified by the appearance of black holes when the cells are plated on fluorochrome-conjugated gelatin matrices. Neutrophils were thus plated onto coverslips coated with fluorochrome-conjugated gelatin matrices and stimulated with fMLP (**Figure 4C**). Rap1b^-/-^ neutrophils exhibited increased proteolytic activity, as they created more black holes that were larger in size than those made by WT cells. This was completely rescued by FX11 treatment. Therefore, LDHA activity mediates Rap1b-loss induced extracellular matrix degradation and F-actin protrusions.

### Acidification drives neutrophil invasive phenotype

**Figure 5:**
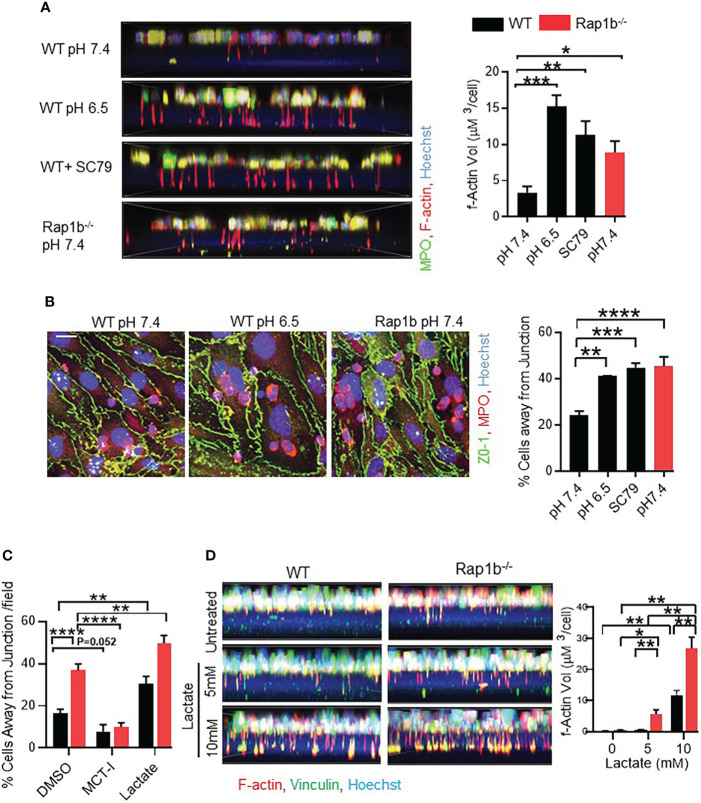
Acidification drives neutrophil invasive phenotype. **(A)** Representative fluorescent images showing formation of protrusions by WT neutrophils after pre-incubation with acidified media (pH6.5) or Akt-Activator (4 μg/mL). Bar graph showing volume quantification of F-actin positive protrusions. (n=minimum 200 cells analyzed/condition/experiment; 3 independent experiments). **(B)** Analysis of neutrophil migration over bEND.3 monolayer. Representative images showing fluorescent staining of fixed neutrophils with MPO (red), ZO-1(green) and Hoechst (blue) after pre-incubation of neutrophils in acidified medium, or with the Akt activator SC79. Bar graph showing quantification of percentage cells away from junction. (n=3 independent experiments, average of 10 fields under 60x objective per experiment). **(C)** Analysis of neutrophil migration over bEND.3 monolayer after pre-incubation of neutrophils with MCT-I, DMSO or after pre-treatment of BEND3 cells in presence of 10mM extracellular lactate. Bar graph showing quantification of percentage cells away from junction. (n=3 independent experiments, average of 10 fields under 60x objective per experiment). **(D)** Representative fluorescent images showing formation of protrusions by WT and Rapb1^-/-^ neutrophils in presence of indicated extracellular lactate in the lower chamber of the transwell. Bar graph showing volume quantification of F-actin positive protrusions. (n=minimum 5 fields under 60x objective, with minimum 200 cells analyzed/condition/experiment; 1 of 2 independent experiments). Mean ± SEM; *P < 0.05; **P < 0.001; ***P < 0.0004; ****P<0.0001, using unpaired Student’s t test. Scale bar; **(A–C)**= 10µm.

Extracellular acidosis has been shown to induce neutrophil activation *via* activation of PI3-K/Akt dependent pathways (21–23). Additionally, at lower pH, neutrophils display increased migration, F-actin polymerization, expression of adhesion promoting receptor, CD18 and delay apoptosis (23). We tested whether acidification can drive WT neutrophil invasive phenotype as seen in Rap1b^-/-^ neutrophils. Extracellular acidification increased intracellular pH in WT neutrophils (***Online*** **Figure S5**). WT neutrophils were incubated in acidified medium (pH 6.5) in 1µm transwell filters for 15 min. Compared to physiological pH, WT neutrophils formed 3-fold more protrusions at pH 6.5 (**Figure 5A**). Protrusion formation also increased in the presence of an Akt-activator. In coculture over LPS-activated bEND3 monolayer, pretreatment of neutrophils with acidic milieu or with Akt activator increased the number of WT neutrophils migrating away from the EC junction (**Figure 5B** ***)***. Blocking neutrophil lactate export using membrane carboxylate transporter inhibitor (MCT-I) α-cyano-4-hydroxycinnamate (CHC) resulted in decrease in neutrophil transcellular migration (**Figure 5C** *)*. On the other hand, addition of 5 or 10 mM extracellular lactate during neutrophil migration onto Bend3 cells increased their transcellular migration (**Figure 5C** *)*. Interestingly, in transwell assay, addition of extracellular lactate to the lower chamber of transwell was sufficient to stimulate protrusion formation of WT neutrophils in absence of chemokine stimulation (**Figure 5D** *)*. Together these results suggest that milieu acidity is sufficient to reprogram WT neutrophils that are more migratory and have increased protrusions, as seen in Rap1b^-/-^ neutrophils.

### Acidification during ischemia reperfusion injury increases vascular permeability and inflammatory outcome *in vivo*

**Figure 6:**
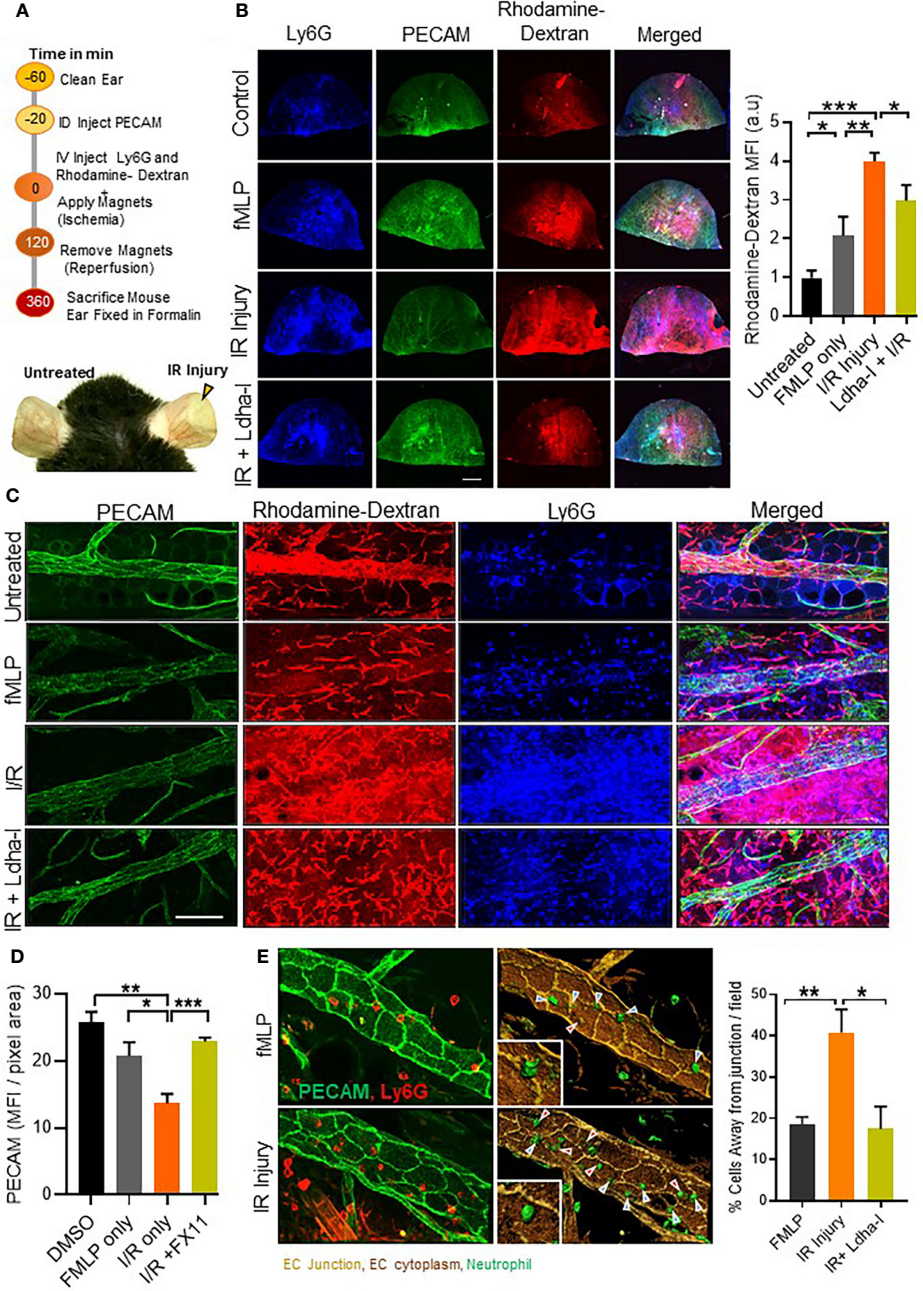
Neutrophil migration and vascular leakage in I/R injury. **(A)** Schematic illustration showing methodology adopted to induce I/R injury using gold-plated, N42-grade neodymium magnets in WT mouse ear vasculature. Representative image (bottom) of mouse ear, showing zone of I/R injury in the right ear, immediately after 2 h of ischemia and left ear without any treatment. **(B)**, Representative fluorescent images of formalin fixed whole ear vasculature showing Pecam-1 stained ear vasculature, Ly6G positive cells and rhodamine-dextran release in presence or absence of treatment as indicated. Bar graph represents fluorescent quantification of rhodamine-dextran release into the ear, normalized to untreated group. (n=4 mice/condition, 4 independent experiments). **(C)**, Merged 2 fields under 60x objective showing infiltration of Ly6G positive cells (blue), rhodamine-dextran release (red) and Pecam-1 (green) staining in ear vasculature after indicated treatment. **(D)**, Bar graph (left) showing quantification of Pecam-1 fluorescence in ear vessels using confocal z-stack images. (n=8 fields under 60x objective were analyzed, 1 of 2 independent experiments). **(E)** Representative 3D reconstituted images showing *in vivo* neutrophil transmigration, built over immunofluorescent images of ear vessel stained with anti-Pecam-1 (pseudo colored to light brown), endothelial cells body (dark brown) and neutrophils (green) using surface module of Imaris software. Cells away from junction (red arrows) and cells at the junction (blue arrows). Bar graph shows percentage of neutrophils migrating away from junction. (n=minimum 6 areas under 60x objective were analyzed, 1 of 2 independent experiment. Red arrow points to transcellular migration events. Mean ± SEM; *P < 0.05; **P < 0.001; ***P < 0.0004; using unpaired Student’s t test. Scale bar; **(B)**=2mm, **(C)**=50µm, **(D)**= 20µm.

Ischemic stroke is the second leading cause of mortality and disability in humans worldwide (24). Local acidification during ischemia has been reported in several animal models (25–27). To examine the role of milieu acidity on neutrophil functions and inflammation *in vivo*, we used an established I/R injury in the ear microvasculature with gold-plated, N42-grade neodymium magnets, as described previously (17) (**Figure 6A**). First, we detected higher rhodamine-dextran leakage in the ear of I/R injury mice compared to fMLP treated group (**Figure 6B**). This was associated with decreased expression of EC junctional protein Pecam-1 in the I/R group compared to the untreated ear or fMLP treated group, and an increased tissue infiltration of Ly6g positive neutrophils (**Figures 6C, D**). Using image analysis tool (Imaris), we further detected increased frequency of neutrophil transmigrating away from EC junction, in I/R injury mice compared to fMLP treated mice (**Figure 6E**). This phenotype was suppressed by FX-11 treatment, suggesting that milieu acidity alters neutrophil/EC interactions and perhaps increases transcellular neutrophil migration at the inflammatory site. Together these results suggest that local tissue acidification increases neutrophil tissue infiltration and vascular injury during I/R.

## Discussion

Excessive neutrophil infiltration into tissue causes inflammatory disorders, such as rheumatoid arthritis and ischemia reperfusion injury (28, 29). Anti-neutrophil antibodies (30) or neutrophil-depleting agents (31) have been successful in reducing ischemia reperfusion injury, at least in animal models. However, we still lack complete understanding of neutrophil migration mechanisms, and how it impacts inflammation. Here, we uncovered that neutrophil acidity *via* Ldha activity increases neutrophil migration *in vivo* and *in vitro*, alters neutrophil-endothelial cell interactions and increases metalloproteinase activity, which likely contribute to increased inflammation. Hence, neutrophil functions are metabolically regulated and alter inflammatory outcome.

In recent years, an increasing body of evidence highlights the importance of cross-talk between cell metabolism and immune response (32–34). It is now recognized that profound changes in tissue metabolism occur at the site of inflammation. These changes can be in the tissue microenvironment, including local depletion of nutrients, increased local acidity (35), or can be immune cell-intrinsic where various metabolite intermediates can directly instruct effector cell functions beyond providing energy. Cellular metabolism is usually the result of a complex interplay between glycolysis and mitochondrial oxidative phosphorylation (OXPHOS). A shift between glycolysis and OXPHOS is well documented to happen in cancer cells and immune cells, including lymphocytes and macrophages, for efficient cell function (36). However, neutrophils are known to exclusively use glycolysis as source of energy and do not rely on OXPHOS (33). We found that stimulation of Rap1b^-/-^ neutrophils with fMLP increased intracellular acidification, which is necessary for their migratory behavior (**Figures 1**, **2**). Our study greatly adds to recent studies showing that chemotactic migration of granulocytes is driven by accelerated uptake of exogenous glucose, highlighting the importance of metabolism regulation during neutrophil transendothelial migration (37). Further, it was shown that neutrophils activated to form neutrophil extracellular trap (NET) exhibit increased glycolysis and lactate production; and that LDHA activity is required for efficient NET formation (38). Hence, lactate is a major component of neutrophil effector functions.

How a change in neutrophil acidity controls cell migration is an interesting question. Acidity through lactate production could increase neutrophil protease activity and subsequent ECM degradation. Indeed, we show that Rap1b^-/-^ neutrophil gelatin degradation activity is highly dependent on Ldha activity (**Figure 4**). Increased lactate production, which is toxic for the cells, usually results in lactate export in the microenvironment and subsequently its acidification (39). Acidification of the milieu is favorable for the activation of proteases, including metalloproteinases, and therefore facilitates cell migration. Acidification of extracellular milieu also seems to increase neutrophil activation (40) and delay apoptosis. Another possibility is that lactate directly modulates cytoskeleton regulation. The formation of invadopodia-like structures in Rap1b^-/-^ neutrophils, which is a critical event of neutrophil transcellular migration, is completely inhibited by inhibition of LDHA activity (**Figure 4**). In support of this, acidification has recently been shown to increase pseudopods-like formation leading to faster chemotactic migration (23). A direct effect on cytoskeleton remodeling protein activity is possible. In cancer cells, Ldha is enriched with actin in the invadopodial structures of cancer cells (41), and induces post-translational modification of cytoskeletal structure protein. Lactic acid can also induce significant neutrophil adhesion reaction onto vascular endothelium in a CD11b/ICAM-1 dependent manner (42). Hence, lactate could act as an underlying biophysical molecular machinery to facilitate the formation of invasive protrusions and transcellular migration.

An important remaining issue will be to determine whether these specific neutrophil-endothelial cell interactions merely control neutrophil transmigration behavior or more globally alter the outcome of inflammation. Here we show that acidity increases neutrophil migration, increases extracellular matrix degradation activity, modifies neutrophil/EC interactions and increases vascular leakage (**Figures 1**-**3**). However, whether these events are linked into a causal/effect relationship remains to be established. The relationship between neutrophil migration and vascular leakage is very contentious. While neutrophils open EC junctions during paracellular migration, there are no indication that this causes vascular leakage (43). In fact, Petri et al. reported that endothelial cells formed a ‘dome’ engulfing leukocytes during paracellular migration, which is thought to minimize vascular leakage (44). During transcellular migration, since the endothelial cell junctions remain intact, increase in vascular permeability is not expected. On the other hand, an increase in extracellular matrix degradation activity, which is necessary for transcellular migration, could affect the vasculature and create tissue damage. A decrease in EC junctional protein expression could also compromise the vasculature (**Figure 6**). Decreased expression of junctional adhesive molecules is known to occur in tissue during inflammatory conditions (7). Interestingly, our study is consistent and adds new findings to a recent report showing that bone marrow neutrophils release lactate in response to lipopolysaccharides or *Salmonella* Typhimurium (45). In this case, lactate released is due to increased glycolysis and NADPH-oxidase mediated reactive oxygen species. In the bone marrow, increased neutrophil lactate is also associated with increased BM vascular permeability by reducing VE-Cadherin expression. Lactate administration causes neutrophil mobilization (45). The increased vascular permeability seen during Rap1b-/- neutrophil migration *in vivo* (**Figure 3**, **6**) could arise from additional effects on inflammation such as cytokine release. Finally, we will need to establish whether acidic-dependent neutrophils represent normal or pathogenic neutrophils. The differential response of WT and Rap1b-/- neutrophils to FX11 suggest that Rap1b-/- neutrophil functions are abnormal and are highly dependent on or ‘addicted to’ LDHA activity, perhaps defining a metabolic addiction state that may be seen in pathogenic but not normal inflammation. This will need to be investigated further.

During wound healing and tumor development the level of lactate in tissue rises several folds under normal physiological conditions (46–48). Lactate is a biomarker of sepsis (49, 50), as its expression drastically increase in septic shock associated with lactic acidosis. Our study suggests that lactate may carry significant role in the pathogenesis of inflammation. Ldha may provide an excellent target for pharmacological intervention. Humans with inherited deletion of 20-bp Ldha gene, had relatively mild symptoms of exertional myopathy (51, 52). Moreover, several Ldha inhibitors are already in clinical trials for their anticancer activity (53), and have been shown to dose-dependently inhibit cancer cells that are dependent on glycolysis (54). Interestingly, Rap1b^-/-^ neutrophils were particularly sensitive to Ldha inhibition such that extracellular matrix degradation activity of Rap1b^-/-^ neutrophils was inhibited at concentration that did not affect WT cells (**Figure 4**). These observations suggest the existence of a therapeutic window to target inflammatory glycolytic neutrophils while leaving intact neutrophils that are less glycolytic, and offer new approaches for specificity in targeting inflammation.

## Data availability statement

The proteomics data presented in the study are deposited in the MassIVE repository, accession number MSV000090706, https://doi.org/doi:10.25345/C5SJ19W3C.

## Author contributions

CS designed and performed experiments, analyzed the data and wrote the paper. EW performed experiments. JX performed experiments. SK provided key advice in research design and helped in experimental design. MK provided key advice in research design, data analysis. MC and SL contributed by providing Rap1b knock out mouse. M-DF designed and directed the program research, analyzed data, and wrote and edited the manuscript. All authors contributed to the article and approved the submitted version.

## Funding

The work was supported by National Institutes of Health (GM112792 to M-DF; HL111582 to MC).

## Acknowledgments

We thank the proteomics facility at Ohio State University, Ohio for LCMSMS orbitrap proteomics analysis. We thank the mouse core, Jeff Bailey and Victoria Summey, for BM transplantation at Cincinnati Children’s Hospital Medical Center.

## Conflict of interest

The authors declare that the research was conducted in the absence of any commercial or financial relationships that could be construed as a potential conflict of interest.

## Publisher’s note

All claims expressed in this article are solely those of the authors and do not necessarily represent those of their affiliated organizations, or those of the publisher, the editors and the reviewers. Any product that may be evaluated in this article, or claim that may be made by its manufacturer, is not guaranteed or endorsed by the publisher.

## Abbreviations

2DG, 2-deoxy-D-glucose; Akt-A, Akt activator; Akt-I, Akt inhibitor; Arp3, Actin-Related Proteins 3; ATP, adenosine tri-phosphate; BAL, bronchoalveolar lavage; bEND.3, brain microvascular endothelial cell 3; BM, bone marrow; DMSO, Dimethyl sulfoxide; EC, endothelial Cell; ECM, extracellular matrix; fMLP, N-Formylmethionyl-leucyl-phenylalanine; G6pd, glucose-6-phosphate dehydrogenase; HBSS, hanks’ balanced salt solution; Hk1, hexokinase-1; I/R, ischemia-reperfusion ; ICAM-1, intercellular adhesion molecule 1; Ldha, lactate dehydrogenase A; Ldha-I, lactate dehydrogenase A inhibitor; LPS, lipopolysaccharides; MMP, matrix metalloproteinases; MPO, Myeloperoxidase; OXPHOS, oxidative phosphorylation; Pecam-1, platelet endothelial cell adhesion molecule 1; PFA, paraformaldehyde; Pgk, phosphoglycerate kinase; PI3-K, phosphatidylinositide 3-kinases; Pkm2, pyruvate kinase isozymes M2; Rap1b, ras-related protein Rap-1b; ROS, reactive oxygen species; WT, wild type; ZO-1, zonula occludens-1.
