# Supplementary material for: Rap1b-loss increases neutrophil lactate dehydrogenase activity to enhance neutrophil migration and acute inflammation in vivo
Source: Front Immunol. 2022 Nov 25;13:1061544. doi: 10.3389/fimmu.2022.1061544 (PMC9733537; doi:10.3389/fimmu.2022.1061544)
Supplement: Supplementary file 1 [file DataSheet_1.pdf]

Supplemental Material

Methods

Chemotaxis assay Chemotactic migration was recorded in transwell chambers as described previously[1], using 3.0 µm transwell inserts coated with 25 µg/mL fibrinogen . Briefly, 5 x 10^5 neutrophils were diluted in HBSS buffer and added to the upper chamber. In the same HBSS buffer, 10µM fMLP was added to induce chemotaxis from lower chamber. Neutrophil migration toward fMLP was allowed for 3 h, at 37ºC. The migrated cells were recovered from the bottom well and counted using hemocytometer. When indicated, neutrophils were pretreated with 0.8 - 4 µM of FX11 or vehicle control DMSO for 45 min before fMLP chemotaxis on transwell filters.

In-vitro Crawling / transendothelial migration (2D Migration) Assay When indicated, neutrophils were pretreated for 45min with either 0.2 µM of Ldha-I (FX11), 4µg/ml Akt activator (SC79), 1mM glycolysis inhibitor (2-DG), or vehicle control DMSO before 10 µM fMLP stimulation. F-actin depolymerization was measured using MFI of F-actin per field per 100 neutrophils using ImageJ software. The paracellular and transcellular route of TEM was identified by immunofluorescence staining with Alexa 488 conjugated ZO-1 antibody to visualize endothelial junctions, anti-MPO antibody to mark neutrophils, F-actin to visualize the EC morphology or with ICAM-1 as well as the membrane remodeling in response to neutrophil adhesion and transmigration.

In vivo transmigration and vascular leakage. Both ears of mice were cleaned with hair removal cream 2 h before experiment. Intraperitoneal injection of 2% (vol/vol) DMSO or 3µg of FX11 (0.15mg/kg body weight) dissolved in PBS was given 1 h before fMLP stimulation. Following incubation, intradermal injection of a mixture containing 10mM fMLP in PBS containing 1 mM Ca2+ and 1 mM Mg2+ along with 0.2µg of Alexa 488-conjugated PECAM antibody was given to stimulate and visualize ear microvasculature. After 4 h of stimulation, Dextran, Tetramethylrhodamine, 70,000 MW, Neutral (50mg/kg body weight) was injected via retro-orbital injection and incubated for another 6 h. Following incubation, mice were sacrificed and both ears were removed and imaged using confocal microscopy after overnight fixation in 10% formalin. For identification of dextran leakage, the whole ear was imaged by taking large Z-stack mosaics (5 × 5 fields) under 4x objective with step size of 25µm were captured with an open pinhole on a Nikon A1R confocal laser scanning microscope with 15% overlap. Florescence quantification was performed using ImageJ software after merging Z-stacks images into a single plane using NIS analysis software. For test cell intrinsic effect of neutrophil Ldha pathway in vivo, neutrophils from WT and Rap1b KO mice were isolated and treated with FX11 or DMSO. Cells were labeled with cell tracker dye and adoptively transferred to recipient mice with FMLP stimulated and Pecam-1 stained ear vasculature. After 2 h ears were collected and fixed in formalin.

Competitive in vivo migration Assay One day before the experiment, dorsal and ventral hair around the ear of albino mice of C57 background was depilated using hair remover cream and rinsed with water. Ear vasculature was stimulated with intradermal injection of

10µm fMLP. Alexa 488-conjugated 15 X10^6 isolated BM neutrophils from WT or Rap1b$^{-/-}$ were labeled with chloromethyl SNARF-1 acetate (CellTracker red; 5 µM; Invitrogen). Either Rap1b$^{-/-}$ or WT received Hoechst stain at 37°C for 10 min. The labeled cells were mixed at a 1:1 ratio and transferred into albino mice through retro-orbital injection. The relative amounts of labeled neutrophils in the circulation after transfer were tested by flow cytometry (FACSCanto; BD; Kumar et al., 2012) of whole blood, withdrawn after 2 h of adoptive transfer. After 10 h of fMLP stimulation, mice were sacrificed and ears were removed. Confocal Z-stack microscopy, under 60x objective was performed after overnight fixation in 10% formalin. Relative neutrophil recruitment was calculated as the ratio of CellTracker red-positive; Hoechst-positive to CellTracker red; Hoechst-negative cells. When indicated, mice were given intraperitoneal injection of DMSO or FX11 (0.15mg/kg body weight) dissolved in PBS, 1 h before fMLP stimulation in the ear.

Ear Ischemia Reperfusion (I/R) Injury –Dorsal hair around ear was removed 2 h before experiment. One hour post injection, mice were anesthetized with ketamine and xylazine anesthesia and intradermal injection of Alexa 488-conjugated PECAM antibody in PBS was given to mark ear vasculature. Intraperitoneal injection of 2% (vol/vol) DMSO or FX11 (1 mg/kg body weight) dissolved in PBS. After 20 min, mixture of fixable Dextran Tetramethylrhodamine 70,000 MW (50mg/kg body weight) and Brilliant Violet 421™ anti-mouse Ly-6G antibody was injected via retro-orbital injection to mark vascular leakage and neutrophil migration respectively. Mice were transferred to an anesthetic chamber and gold-plated N42-grade neodymium magnets placed at the outer edge one of the ears. After 2 h incubation under anesthesia, magnets were removed carefully to allow

reperfusion to take place for 2 to 4 h. Finally, ears were collected after sacrificing the mice, formalin fixed overnight at RT and imaged under confocal microscope.

Bioinformatics Scaffold Proteome Software Inc. (version 4.4.5) was used to validate MS/MS based peptide and protein identifications. Peptide identifications were accepted if they could be established at greater than 95.0% probability to achieve an FDR less than 1.0% by the Peptide Prophet algorithm 95 with Scaffold delta-mass correction. Protein identifications were accepted if they could be established at greater than 100% probability to achieve an FDR less than 1.0% and contained at least 5 identified peptides. Protein probabilities were assigned by the Protein Prophet algorithm[2]. Proteins with minimum of 25% change protein expression in Rap1b-/- compared to WT were selected for each kinetic point. GO cellular component ontology and pathway clustering was performed using Toppcluster online application. Bonferroni correction and p-value cutoff of 0.05 was applied. The generated network map was exported to Cytoscape (version 3.2.1) and diagram was drawn based on enrichment analysis performed with ToppCluster.

Image acquisition and processing Confocal imaging was performed with Nikon A1R confocal laser scanning microscope (Nikon) using a 4x, 20x, and 60x objectives. Image processing including background subtraction, 3D reconstruction, and projection of Z-stacks were performed with NIS-elements (version 4.5) image analysis software or Imaris (version 9.0). Images were then exported in JPG or TIFF format and corrected total cell

fluorescence (CTCF) or mean fluorescence intensity (MFI) was measured using ImageJ (version 1.51k).

	Antibodies	Description	Catalogue	Company
1	Vinculin	Mouse Monoclonal Anti-Vinculin. Clone VIN-11-5	V4505	Sigma-Aldrich
2	MPO	Rabbit Polyclonal Anti-Human Myeloperoxidase	A0398	Dako
3	Pkm2	Rabbit monoclonal PKM2 (D78A4) XP®	4053	Cell Signaling
4	Ldha	Mouse monoclonal LDH-A Antibody (E-9)	sc-137243	Santa Cruz
5	β-Actin-HRP	Anti-beta Actin antibody [AC-15] (HRP)	ab49900	Abcam
6	CD31 / PECAM-1	Alexa Fluor® 488 anti-mouse CD31, Clone 390	102413	BioLegend
7	Hexokinase1	Rabbit monoclonal Hexokinase I (C35C4)	2024	Cell Signaling
8	G6PD	Rabbit Polyclonal G6PD antibody	A300-404A	Bethyl Lab
9	CD44	Rat Anti-Mouse CD44 antibody, Clone IM7 (RUO)	550538	BD Biosciences
10	Arp2	Mouse monoclonal Arp2 Antibody (E-2)	sc-137250	Santa Cruz
11	Myosin 2b	Rabbit monoclonal Myosin IIb (D8H8) XP® Antibody	8824	Cell Signaling
12	Rap1b	Rap1B (36E1) Rabbit monoclonal Antibody	2326	Cell Signaling
13	ZO-1	ZO-1 Monoclonal Antibody (ZO1-1A12), Alexa Fluor 488	339188	Fisher Scientific
	**Chemicals**	**Description**	**Catalogue**	**Company**
1	pHrodo	Invitrogen pHrodo Red 10,000 MW Dextran	P35372	ThermoFisher
2	2NBDG	(2-(N-(7-Nitrobenz-2-oxa-1,3-diazol-4-yl)Amino)-2-Deoxyglucose	N13195	ThermoFisher
3	LPS	Lipopolysaccharides from Escherichia coli O26:B6	L2654	Sigma-Aldrich
4	fMLP	N-Formyl-Met-Leu-Phe	F3506	Sigma-Aldrich
5	Gelatin matrix	Gelatin From Pig Skin, Oregon Green™ 488 Conjugate	G13186	ThermoFisher
6	Fibrinogen	Fibrinogen from murine plasma	F3879	Sigma-Aldrich
7	Ldha-Inhibitor	Lactate Dehydrogenase A Inhibitor, FX11	427218	Calbiochem
8	Akt-Inhibitor	MK-2206 2HCL	S1078	Selleck Chemicals
10	MCT-Inhibitor	α-Cyano-4-hydroxycinnamic acid	C8982	Sigma-Aldrich
11	Glycolysis Inhibitor	2-Deoxy-D-glucose	D8375	Sigma-Aldrich
12	Dextran	Dextran, Tetramethylrhodamine, 70,000 MW, Neutral	D1819	Life Technologics
13	Bend.3 Media	DMEM ATCC	30-2002	ATCC
14	Mounting Media	SlowFade Diamond Antifade Mountant	S36963	Molecular probes
15	Actin philloidin	Rhodamine phalloidin	R415	Life Technologics
16	LdhaHI	Lactate Dehydrogenase Inhibitor II, GSK2837808A	533660	Millipore
17	Akt-Activator	Akt Activator II, SC79 - CAS 305834-79-1	123871	Millipore
18	Histopaque	Histopaque®-1119 and Histopaque®-1077	11191/ 10771	Sigma
19	Cell Lysis buffer	RIPA Lysis and Extraction Buffer	89900	TermoFisher
	**Kits / Supplies**	**Description**	**Catalogue**	**Company**
1	Total Protein	Pierce BCA Protein Assay Kit	23227	Fisher Scientific
2	Albumin assay kit	BCG Albumin Assay Kit	MAK124	Sigma-Aldrich
3	Substrate for WB	SuperSignal™ West Pico PLUS Chemiluminescent Substrate	34580	ThermoFisher
4	Transwell 1.0um	Falcon® Permeable Support for 6 Well Plate with 1.0µm Transparent PET Membrane, Sterile, 1/Pack, 48/Case	353102	Corning
5	Transwell 3.0uM	24 mm Transwell with 3.0 µm pore polycarbonate membrane insert, TC-treated, w/lid, sterile, 24/cs	CLS3414	Sigma-Aldrich
6	Lactate assay kit	Lactate Dehydrogenase A/LDHA Assay Kit (Colorimetric)	KA0786	Novus Biologicals
7	ATP assay kit	ATP Determination Kit	A22066	TermoFisher

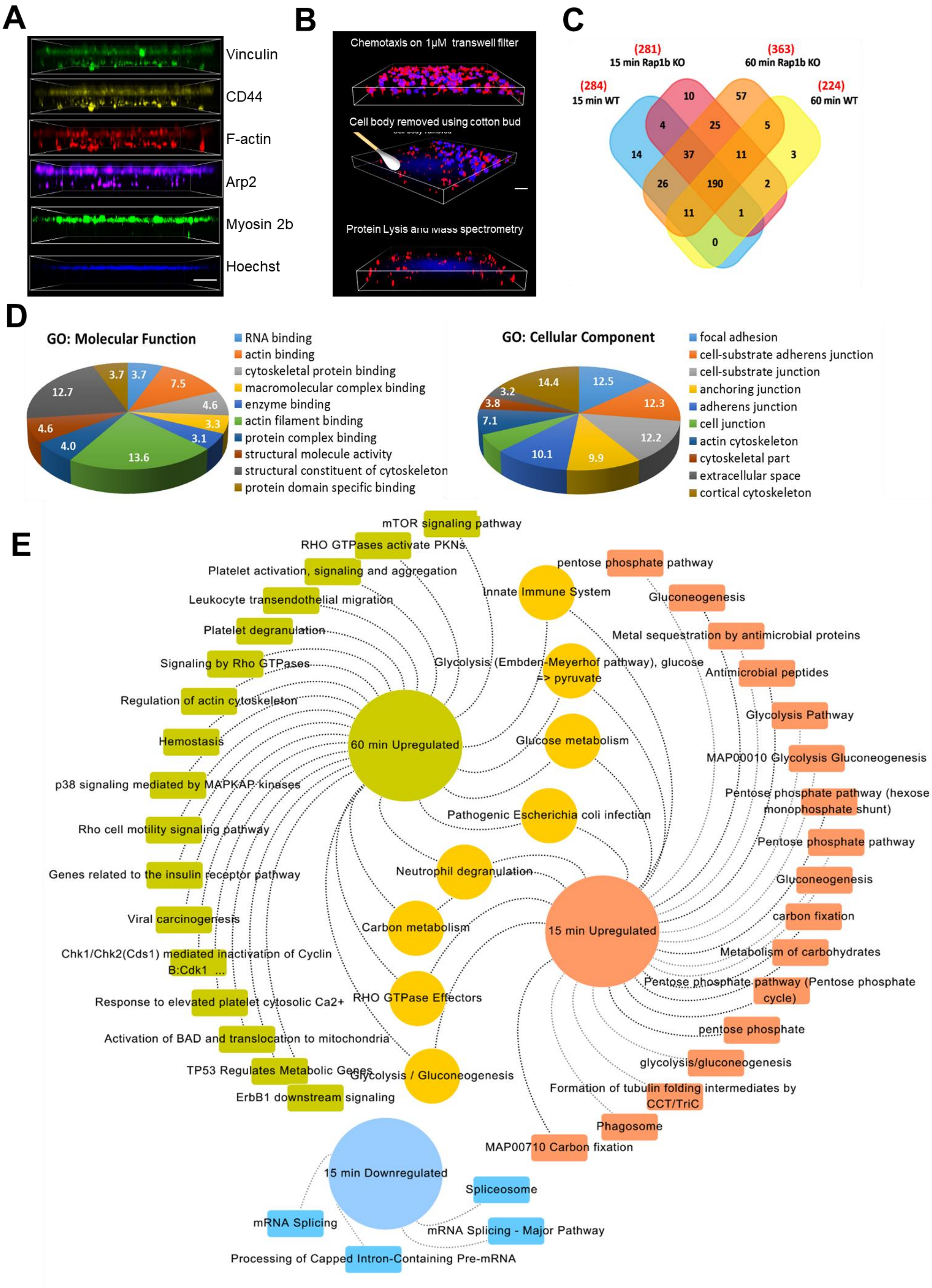
A
Vinculin
CD44
F-actin
Arp2
Myosin 2b
Hoechst
B
Chemotaxis on 1μM transwell filter
Cell body removed using cotton bud
Protein Lysis and Mass spectrometry
C
(281)
15 min Rap1b KO
(363)
60 min Rap1b KO
(284)
15 min WT
(224)
60 min WT
10
57
4
25
5
14
37
11
3
26
190
2
11
1
0
D
GO: Molecular Function
RNA binding
actin binding
cytoskeletal protein binding
macromolecular complex binding
enzyme binding
actin filament binding
protein complex binding
structural molecule activity
structural constituent of cytoskeleton
protein domain specific binding
3.7
3.7
7.5
4.6
3.3
3.1
13.6
4.0
4.6
12.7
GO: Cellular Component
focal adhesion
cell-substrate adherens junction
cell-substrate junction
anchoring junction
adherens junction
cell junction
actin cytoskeleton
cytoskeletal part
extracellular space
cortical cytoskeleton
12.5
12.3
12.2
9.9
10.1
7.1
3.8
3.2
14.4
E
mTOR signaling pathway
RHO GTPases activate PKNs
Platelet activation, signaling and aggregation
Leukocyte transendothelial migration
Platelet degranulation
Signaling by Rho GTPases
Regulation of actin cytoskeleton
Hemostasis
p38 signaling mediated by MAPKAP kinases
Rho cell motility signaling pathway
Genes related to the insulin receptor pathway
Viral carcinogenesis
Chk1/Chk2(Cds1) mediated inactivation of Cyclin B:Cdk1 ...
Response to elevated platelet cytosolic Ca2+
Activation of BAD and translocation to mitochondria
TP53 Regulates Metabolic Genes
ErbB1 downstream signaling
60 min Upregulated
Innate Immune System
Glycolysis (Embden-Meyerhof pathway), glucose => pyruvate
Glucose metabolism
Pathogenic Escherichia coli infection
Neutrophil degranulation
Carbon metabolism
RHO GTPase Effectors
Glycolysis / Gluconeogenesis
15 min Upregulated
pentose phosphate pathway
Gluconeogenesis
Metal sequestration by antimicrobial proteins
Antimicrobial peptides
Glycolysis Pathway
MAP00010 Glycolysis Gluconeogenesis
Pentose phosphate pathway (hexose monophosphate shunt)
Pentose phosphate pathway
Gluconeogenesis
carbon fixation
Metabolism of carbohydrates
Pentose phosphate pathway (Pentose phosphate cycle)
pentose phosphate
glycolysis/gluconeogenesis
Formation of tubulin folding intermediates by CCT/TriC
Phagosome
MAP00710 Carbon fixation
15 min Downregulated
Spliceosome
mRNA Splicing
mRNA Splicing - Major Pathway
Processing of Capped Intron-Containing Pre-mRNA

Online Figure S1- Visualization, isolation and mass spectrometry of neutrophil protrusions – **A**, Fixed neutrophils on 1µM transwell filter after 60min of fMLP chemotaxis and stained with Vinculin (green) and F-actin(red), CD44(yellow), Arp3(purple), Myosin2b (green) and Hoechst (blue). **B**, Schematic illustration using Z-stack confocal images, showing removal of neutrophil cell body from top of 1µm transwell filters, to enrich protrusion fraction for mass spectrometry. Staining F-actin (red), Hoechst (blue). **C**, Venn diagram showing overlap between protrusion proteins of 4 groups WT (15min and 60min) and Rap1b$^{-/-}$ (15min and 60min) which are above 5 peptide threshold, as identified by mass spectrometry. **D,** GO analysis showing of Cellular component and Molecular function of 190 proteins present in all 4 sample groups, based on p-Value. Pie charts indicate the percent of identified genes from input in respective Go category listed in the legend. **E,** 25 most significant enriched pathways based on p-value scoring, are listed and compared between 15min and 60min fractions. Common pathways are highlighted with yellow. Visualization was generated using Cytoscape software. Scale bar; (A,B)=10µm.

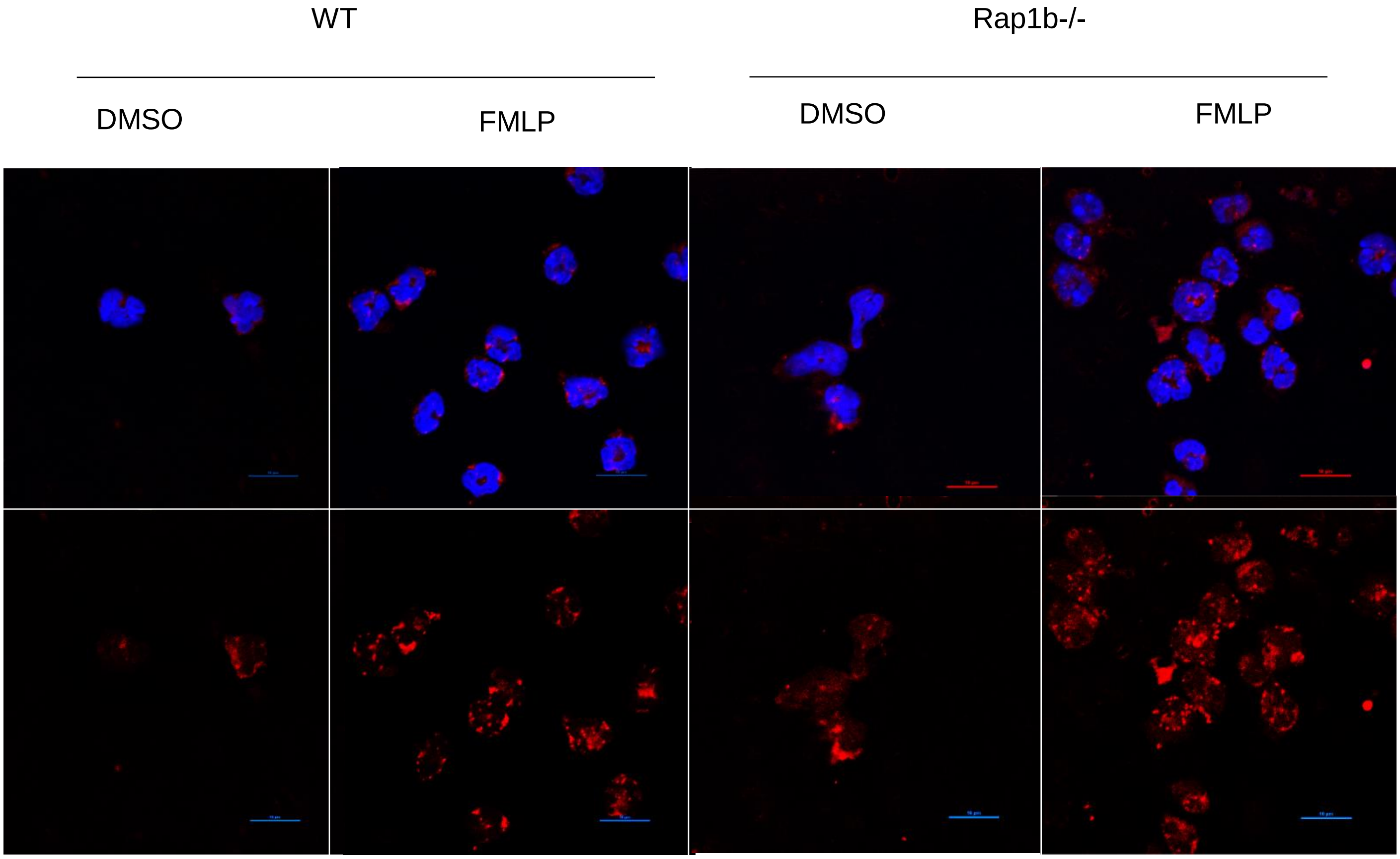

Online Figure S2 images of intracellular neutrophil pH

Representative images showing region of intracellular acidification by pH sensitive dye, pHrodo, in WT and Rap1b$^{-/-}$ neutrophils before and after 15min of fMLP stimulation. Red is pHrodo, blue is dapi. Scale bar is 10μm

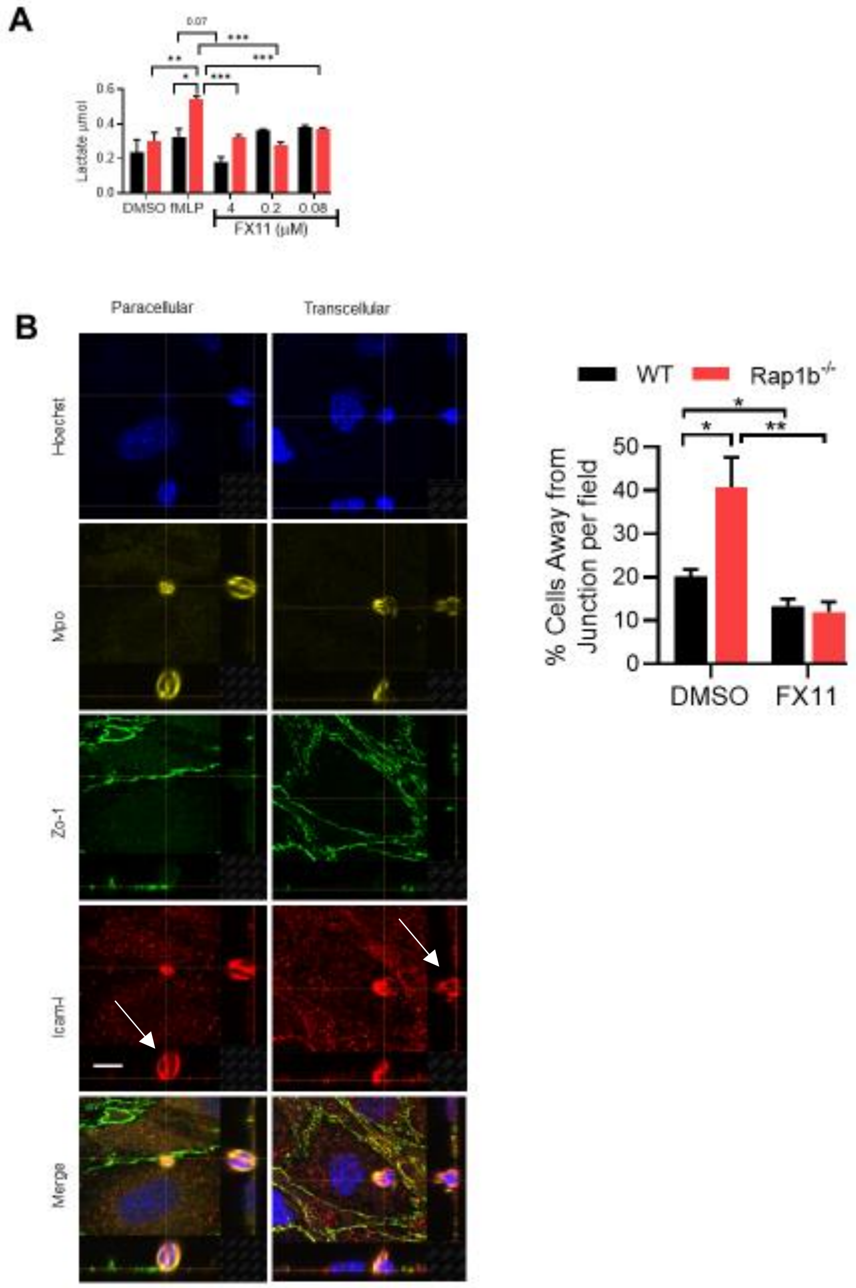

A
0.07
***
**
***
*
***
Lactate µmol
0.6
0.4
0.2
0.0
DMSO fMLP
4
0.2
0.08
FX11 (µM)
B
Paracellular
Transcellular
Hoechst
Mpo
Zo-1
Icam-I
Merge
WT
Rap1b-/-
*
*
**
% Cells Away from Junction per field
50
40
30
20
10
0
DMSO
FX11

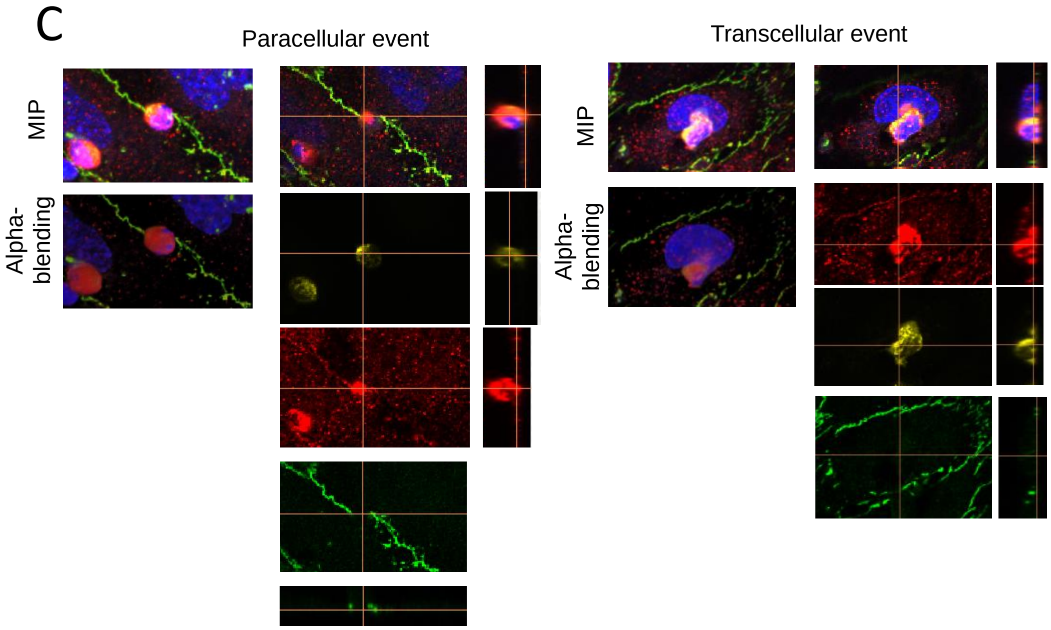
C
Paracellular event
Transcellular event
MIP
Alpha-blending
MIP
Alpha-blending

Online Figure S3- A, Quantification of Lactate release by adherent neutrophils after fMLP stimulation and treatment with FX-11 (n=4 mice per group per condition. 2 independent experiments). **B**, Representative immunofluorescence images showing paracellular and transcellular migration of neutrophils, identified using ZO-1, ICAM-1, and MPO and nuclear staining. The white arrow shows the ring of ICAM on the Z plan. Bar graph showing percentage of WT and Rap1b$^{-/-}$ neutrophils showing diapedesis via transcellular route in the presence or absence of Ldha Inhibitor FX11. (N=5 independent area under 60x objective. Minimum 30 migration events were counted per group. 1 of 2 independent experiments. Mean ± SEM; *, $P < 0.05$; ***, $P < 0.0004$; using unpaired Student's t test. Scale bar; (B,C)= 10μm. **C.** Additional representative images of paracellular and transcellular migration showing maximum intensity projection (MIP) and alpha-blending views. Note the cell on the right panel which is in the act of migration away from the junction.

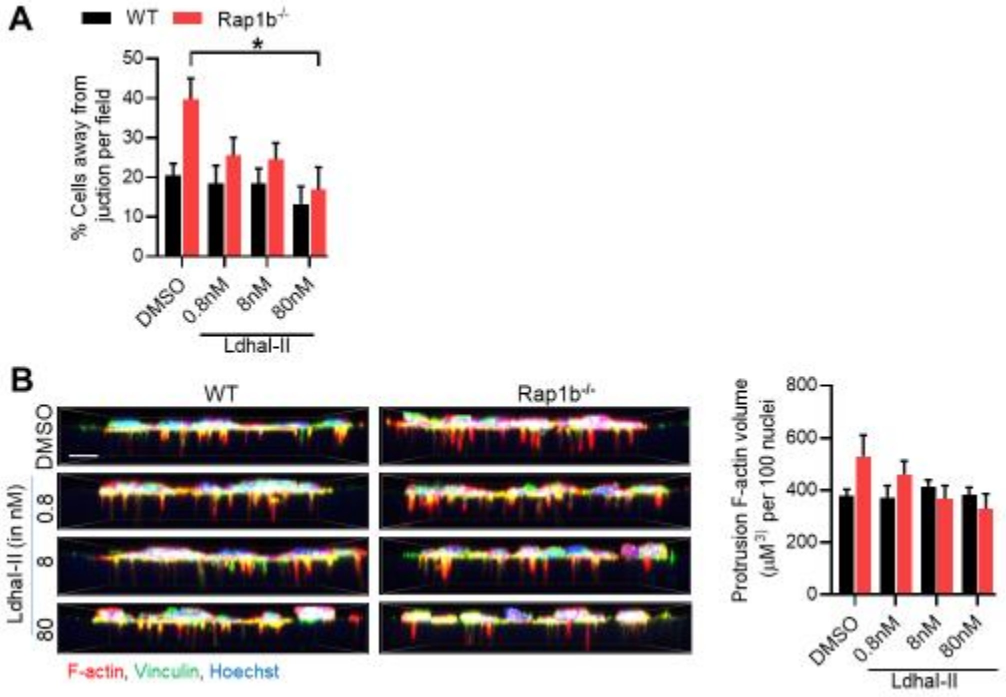

Online figure S4- Effect of LdhaI-II on transendothelial migration and F-actin polymerization– **A**, Bar graph showing percentage of cells migrating away from junction per field in 2D migration assay. Minimum n=5 fields under 60x objective were counted for each data set. 2 independent experiments. **B**, Representative confocal images showing formation of protrusions in 1µM transwell filters by WT and Rap1b$^{-/-}$ neutrophils in the presence or absence of the Ldha inhibitor GSK2837808A (LdhaI-II) at indicated concentration. Bar graph showing quantification of volume of F-actin protrusion captured in the pores of 1µm transwell filters after pretreatment of neutrophils with LdhaI-II using surface module of Imaris software. (n=30 cells analyzed each data set, 1 of 2 independent

experiments). Mean ± SEM; *, P < 0.05; ***, P < 0.0004; ****, P<0.0001, using unpaired Student's t test. Scale bar; (A,B)= 10µm

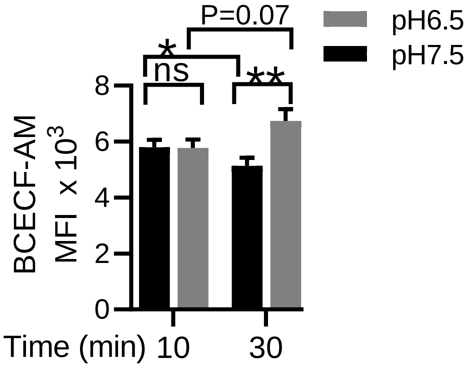

Online Figure S5- Extracellular acidification altered neutrophil intracellular pH. Bar graph showing quantifications of intracellular BCECF fluorescence of neutrophils after incubating cells for 10 min and 30 min in HBSS media at pH 6.7 and 7.5.

Identified Proteins	Peptide detected by MS			
	WT 15min	Rap1b KO 15min	WT 1h	Rap1b KO 1h
Actin, cytoplasmic 2 OS=M+E5:E635us musculus GN=Actg1 PE=1 SV=1	180	237	219	255
Lactotransferrin OS=Mus musculus GN=Ltf PE=2 SV=4	186	218	194	222
Protein S100-A8 OS=Mus musculus GN=S100a8 PE=1 SV=3	97	150	163	95
Protein S100-A9 OS=Mus musculus GN=S100a9 PE=1 SV=3	74	179	122	99
Myeloperoxidase OS=Mus musculus GN=Mpo PE=2 SV=2	104	119	107	141
Histone H4 OS=Mus musculus GN=Hist1h4a PE=1 SV=2	103	88	74	140
Pyruvate kinase PKM OS=Mus musculus GN=Pkm PE=1 SV=4	91	110	80	118
Histone H2B type 1-C/E/G OS=Mus musculus GN=Hist1h2bc PE=1 SV=3	65	102	107	121
Neutrophilic granule protein OS=Mus musculus GN=Ngp PE=1 SV=1	78	110	105	87
Annexin A1 OS=Mus musculus GN=Anxa1 PE=1 SV=2	81	96	94	100
Filamin-A OS=Mus musculus GN=Flna PE=1 SV=5	97	62	97	115
Alpha-enolase OS=Mus musculus GN=Eno1 PE=1 SV=3	73	93	79	91
Talin-1 OS=Mus musculus GN=Tln1 PE=1 SV=2	90	68	72	103
Tubulin beta-5 chain OS=Mus musculus GN=Tubb5 PE=1 SV=1	61	91	59	103
Transketolase OS=Mus musculus GN=Tkt PE=1 SV=1	69	86	69	80
Chitinase-like protein 3 OS=Mus musculus GN=Chil3 PE=1 SV=2	64	91	58	82
Plastin-2 OS=Mus musculus GN=Lcp1 PE=1 SV=4	68	80	57	87
Glyceraldehyde-3-phosphate dehydrogenase OS=Mus musculus GN=Gapdh PE=1 SV=2	59	70	74	84
Hemoglobin subunit beta-1 OS=Mus musculus GN=Hbb-b1 PE=1 SV=2	58	50	48	67
Histone H2A type 1 OS=Mus musculus GN=Hist1h2ab PE=1 SV=3	37	39	75	60
Heat shock cognate 71 kDa protein OS=Mus musculus GN=Hspa8 PE=1 SV=1	57	47	46	59
Tubulin alpha-1B chain OS=Mus musculus GN=Tuba1b PE=1 SV=2	39	56	37	58
Peptidyl-prolyl cis-trans isomerase A OS=Mus musculus GN=Ppia PE=1 SV=2	39	45	46	58
Profilin-1 OS=Mus musculus GN=Pfn1 PE=1 SV=2	46	41	49	49
Cofilin-1 OS=Mus musculus GN=Cfl1 PE=1 SV=3	42	49	41	52
Lysozyme C-2 OS=Mus musculus GN=Lyz2 PE=1 SV=2	31	57	47	44
Vimentin OS=Mus musculus GN=Vim PE=1 SV=3	50	29	32	66
Fructose-bisphosphate aldolase A OS=Mus musculus GN=Aldoa PE=1 SV=2	42	46	40	40
Cathelin-related antimicrobial peptide OS=Mus musculus GN=Camp PE=2 SV=1	33	40	47	41
Neutrophil elastase OS=Mus musculus GN=Elane PE=2 SV=1	33	45	30	35
Integrin beta-2 OS=Mus musculus GN=Itgb2 PE=1 SV=2	32	29	24	56
14-3-3 protein zeta/delta OS=Mus musculus GN=Ywhaz PE=1 SV=1	29	35	32	43
Heterogeneous nuclear ribonucleoproteins A2/B1 OS=Mus musculus GN=Hnrnpa2b1 PE=1 SV=2	35	30	32	40
Rho GDP-dissociation inhibitor 2 OS=Mus musculus GN=Arhgdib PE=2 SV=3	31	38	31	34
Ras GTPase-activating-like protein IQGAP1 OS=Mus musculus GN=Iqgap1 PE=1 SV=2	30	39	21	44
Leukotriene A-4 hydrolase OS=Mus musculus GN=Lta4h PE=1 SV=4	35	38	17	42
Adenylyl cyclase-associated protein 1 OS=Mus musculus GN=Cap1 PE=1 SV=4	29	34	25	38
Glucose-6-phosphate isomerase OS=Mus musculus GN=Gpi PE=1 SV=4	32	27	23	42
Coronin-1A OS=Mus musculus GN=Coro1a PE=1 SV=5	25	28	33	35
Leukocyte elastase inhibitor A OS=Mus musculus GN=Serpinb1a PE=1 SV=1	29	37	19	33

Elongation factor 2 OS=Mus musculus GN=Eef2 PE=1 SV=2	25	33	22	35
Eosinophil peroxidase OS=Mus musculus GN=Epx PE=1 SV=2	34	29	27	45
Alpha-actinin-4 OS=Mus musculus GN=Actn4 PE=1 SV=1	25	21	18	47
6-phosphogluconate dehydrogenase, decarboxylating OS=Mus musculus GN=Pgd PE=1 SV=3	22	31	20	37
Cathepsin G OS=Mus musculus GN=Ctsg PE=1 SV=2	24	31	35	19
Peroxiredoxin-5, mitochondrial OS=Mus musculus GN=Prdx5 PE=1 SV=2	22	29	30	28
Transitional endoplasmic reticulum ATPase OS=Mus musculus GN=Vcp PE=1 SV=4	26	26	22	33
Elongation factor 1-alpha 1 OS=Mus musculus GN=Eef1a1 PE=1 SV=3	29	27	16	34
Gelsolin OS=Mus musculus GN=Gsn PE=1 SV=3	23	26	27	30
78 kDa glucose-regulated protein OS=Mus musculus GN=Hspa5 PE=1 SV=3	29	32	30	33
Phosphoglycerate kinase 1 OS=Mus musculus GN=Pgk1 PE=1 SV=4	17	30	31	28
Myosin light polypeptide 6 OS=Mus musculus GN=Myl6 PE=1 SV=3	23	26	28	27
Vinculin OS=Mus musculus GN=Vcl PE=1 SV=4	19	20	25	37
Histone H3.2 OS=Mus musculus GN=Hist1h3b PE=1 SV=2	30	28	21	20
Heterogeneous nuclear ribonucleoprotein K OS=Mus musculus GN=Hnrnpk PE=1 SV=1	24	22	21	30
Transgelin-2 OS=Mus musculus GN=Tagln2 PE=1 SV=4	17	24	26	27
Malate dehydrogenase, mitochondrial OS=Mus musculus GN=Mdh2 PE=1 SV=3	19	29	14	29
Protein disulfide-isomerase OS=Mus musculus GN=P4hb PE=1 SV=2	24	22	23	21
Hemoglobin subunit alpha OS=Mus musculus GN=Hba PE=1 SV=2	24	18	23	24
Aldehyde dehydrogenase, mitochondrial OS=Mus musculus GN=Aldh2 PE=1 SV=1	17	21	17	31
Myosin regulatory light chain 12B OS=Mus musculus GN=Myl12b PE=1 SV=2	17	28	14	27
Neutrophil gelatinase-associated lipocalin OS=Mus musculus GN=Lcn2 PE=1 SV=1	18	26	22	20
WD repeat-containing protein 1 OS=Mus musculus GN=Wdr1 PE=1 SV=3	21	20	19	26
ATP synthase subunit beta, mitochondrial OS=Mus musculus GN=Atp5b PE=1 SV=2	18	27	13	27
Myeloblastin OS=Mus musculus GN=Prtn3 PE=2 SV=2	13	29	19	24
CD177 antigen OS=Mus musculus GN=Cd177 PE=2 SV=1	25	17	15	26
Lamin-B1 OS=Mus musculus GN=Lmnb1 PE=1 SV=3	22	15	14	31
Matrix metalloproteinase-9 OS=Mus musculus GN=Mmp9 PE=2 SV=2	23	14	15	30
Integrin alpha-M OS=Mus musculus GN=Itgam PE=1 SV=2	18	22	14	27
Glutathione S-transferase Mu 1 OS=Mus musculus GN=Gstm1 PE=1 SV=2	18	21	12	28
Lymphocyte-specific protein 1 OS=Mus musculus GN=Lsp1 PE=1 SV=2	23	18	23	15
Glycogen phosphorylase, liver form OS=Mus musculus GN=Pygl PE=1 SV=4	19	22	12	24
Glucose-6-phosphate 1-dehydrogenase X OS=Mus musculus GN=G6pdx PE=1 SV=3	19	24	7	26
Hexokinase-3 OS=Mus musculus GN=Hk3 PE=2 SV=2	19	26	10	19
Protein disulfide-isomerase A3 OS=Mus musculus GN=Pdia3 PE=1 SV=2	21	13	14	26
Bone marrow proteoglycan OS=Mus musculus GN=Prg2 PE=1 SV=1	23	18	13	18
Alpha-actinin-1 OS=Mus musculus GN=Actn1 PE=1 SV=1	30	30	20	52
Eosinophil cationic protein 2 OS=Mus musculus GN=Ear2 PE=2 SV=1	17	17	20	16
Annexin A2 OS=Mus musculus GN=Anxa2 PE=1 SV=2	12	20	14	23
ATP synthase subunit alpha, mitochondrial OS=Mus musculus GN=Atp5a1 PE=1 SV=1	16	20	13	20
Actin-related protein 3 OS=Mus musculus GN=Actr3 PE=1 SV=3	16	15	13	24
Actin-related protein 2/3 complex subunit 5 OS=Mus musculus GN=Arpc5 PE=2 SV=3	17	16	18	16
Nucleophosmin OS=Mus musculus GN=Npm1 PE=1 SV=1	12	18	18	19

Neutrophil cytosol factor 4 OS=Mus musculus GN=Ncf4 PE=1 SV=2	12	18	14	21
Ubiquitin-like modifier-activating enzyme 1 OS=Mus musculus GN=Uba1 PE=1 SV=1	14	16	12	23
Destrin OS=Mus musculus GN=Dstn PE=1 SV=3	18	18	10	18
Rab GDP dissociation inhibitor beta OS=Mus musculus GN=Gdi2 PE=1 SV=1	13	15	10	26
Heterogeneous nuclear ribonucleoprotein U OS=Mus musculus GN=Hnrnpu PE=1 SV=1	21	10	7	24
Transaldolase OS=Mus musculus GN=Taldo1 PE=1 SV=2	12	18	14	17
Glutathione peroxidase 1 OS=Mus musculus GN=Gpx1 PE=1 SV=2	15	16	15	14
Phosphoglycerate mutase 1 OS=Mus musculus GN=Pgam1 PE=1 SV=3	14	9	14	22
Hematopoietic lineage cell-specific protein OS=Mus musculus GN=Hcls1 PE=1 SV=2	16	10	18	14
Neutrophil cytosol factor 1 OS=Mus musculus GN=Ncf1 PE=2 SV=3	9	20	13	16
Heterogeneous nuclear ribonucleoprotein A3 OS=Mus musculus GN=Hnrnpa3 PE=1 SV=1	16	8	11	23
Peroxiredoxin-6 OS=Mus musculus GN=Prdx6 PE=1 SV=3	14	17	10	16
L-lactate dehydrogenase A chain OS=Mus musculus GN=Ldha PE=1 SV=3	10	21	7	17
ADP/ATP translocase 2 OS=Mus musculus GN=Slc25a5 PE=1 SV=3	12	11	9	22
Ubiquitin-60S ribosomal protein L40 OS=Mus musculus GN=Uba52 PE=1 SV=2	11	11	17	15
Actin-related protein 2/3 complex subunit 1B OS=Mus musculus GN=Arpc1b PE=2 SV=4	12	16	10	14
Guanine nucleotide-binding protein G(I)/G(S)/G(T) subunit beta-2 OS=Mus musculus GN=Gnb2 PE=1 SV=3	8	17	10	17
60S acidic ribosomal protein P0 OS=Mus musculus GN=Rplp0 PE=1 SV=3	13	16	7	16
Tyrosine-protein phosphatase non-receptor type 6 OS=Mus musculus GN=Ptpn6 PE=1 SV=2	12	15	7	17
Transforming protein RhoA OS=Mus musculus GN=Rhoa PE=1 SV=1	13	15	10	13
Annexin A5 OS=Mus musculus GN=Anxa5 PE=1 SV=1	11	12	10	16
Rho GDP-dissociation inhibitor 1 OS=Mus musculus GN=Arhgdia PE=1 SV=3	10	12	10	17
Keratin, type II cytoskeletal 5 OS=Mus musculus GN=Krt5 PE=1 SV=1	13	12	7	17
Protein S100-A11 OS=Mus musculus GN=S100a11 PE=1 SV=1	14	12	12	11
Purine nucleoside phosphorylase OS=Mus musculus GN=Pnp PE=1 SV=2	9	17	6	16
F-actin-capping protein subunit beta OS=Mus musculus GN=Capzb PE=1 SV=3	12	10	8	16
Heterogeneous nuclear ribonucleoprotein M OS=Mus musculus GN=Hnrnpm PE=1 SV=3	11	5	13	17
LIM and SH3 domain protein 1 OS=Mus musculus GN=Lasp1 PE=1 SV=1	13	7	11	14
Perilipin-3 OS=Mus musculus GN=Plin3 PE=1 SV=1	9	12	11	13
Drebrin-like protein OS=Mus musculus GN=Dbnl PE=1 SV=2	11	10	11	12
Ribonuclease inhibitor OS=Mus musculus GN=Rnh1 PE=1 SV=1	13	7	8	16
Nuclease-sensitive element-binding protein 1 OS=Mus musculus GN=Ybx1 PE=1 SV=3	10	11	12	11
Calmodulin OS=Mus musculus GN=Calm1 PE=1 SV=2	9	12	14	8
Poly(rC)-binding protein 1 OS=Mus musculus GN=Pcbp1 PE=1 SV=1	11	7	10	15
Ras-related C3 botulinum toxin substrate 2 OS=Mus musculus GN=Rac2 PE=2 SV=1	14	12	7	10
Fermitin family homolog 3 OS=Mus musculus GN=Fermt3 PE=1 SV=1	13	11	5	14
ADP-dependent glucokinase OS=Mus musculus GN=Adpgk PE=1 SV=2	8	14	6	14
Actin-related protein 2 OS=Mus musculus GN=Actr2 PE=1 SV=1	10	11	8	13
60 kDa heat shock protein, mitochondrial OS=Mus musculus GN=Hspd1 PE=1 SV=1	10	9	8	15
Histone H1.2 OS=Mus musculus GN=Hist1h1c PE=1 SV=2	12	10	12	8
Cell division control protein 42 homolog OS=Mus musculus GN=Cdc42 PE=1 SV=2	9	7	6	20

Heterogeneous nuclear ribonucleoprotein L OS=Mus musculus GN=Hnrnpl PE=1 SV=2	7	10	7	17
Actin-related protein 2/3 complex subunit 4 OS=Mus musculus GN=Arpc4 PE=1 SV=3	8	12	9	11
Complement C3 OS=Mus musculus GN=C3 PE=1 SV=3	11	11	5	12
Annexin A3 OS=Mus musculus GN=Anxa3 PE=1 SV=4	11	13	7	20
F-actin-capping protein subunit alpha-1 OS=Mus musculus GN=Capza1 PE=1 SV=4	7	8	9	14
Glutathione reductase, mitochondrial OS=Mus musculus GN=Gsr PE=1 SV=3	10	11	8	9
Keratin, type I cytoskeletal 10 OS=Mus musculus GN=Krt10 PE=1 SV=3	10	12	5	11
Plasminogen activator inhibitor 1 RNA-binding protein OS=Mus musculus GN=Serbp1 PE=1 SV=2	7	11	9	11
Core histone macro-H2A.1 OS=Mus musculus GN=H2afy PE=1 SV=3	12	5	7	14
14-3-3 protein epsilon OS=Mus musculus GN=Ywhae PE=1 SV=1	15	16	10	18
14-3-3 protein gamma OS=Mus musculus GN=Ywhag PE=1 SV=2	15	14	12	18
40S ribosomal protein SA OS=Mus musculus GN=Rpsa PE=1 SV=4	8	8	8	13
Eukaryotic initiation factor 4A-I OS=Mus musculus GN=Eif4a1 PE=1 SV=1	8	10	7	11
Heterogeneous nuclear ribonucleoprotein A/B OS=Mus musculus GN=Hnrnpab PE=1 SV=1	11	10	5	9
Thioredoxin OS=Mus musculus GN=Txn PE=1 SV=3	12	6	7	10
Chromobox protein homolog 3 OS=Mus musculus GN=Cbx3 PE=1 SV=2	6	8	9	11
Calponin-2 OS=Mus musculus GN=Cnn2 PE=1 SV=1	6	7	9	12
40S ribosomal protein S3 OS=Mus musculus GN=Rps3 PE=1 SV=1	7	9	5	13
Triosephosphate isomerase OS=Mus musculus GN=Tpi1 PE=1 SV=4	7	10	6	11
Eosinophil cationic protein 1 OS=Mus musculus GN=Ear1 PE=2 SV=1	13	13	13	17
Rho-related GTP-binding protein RhoG OS=Mus musculus GN=Rhog PE=2 SV=1	9	10	5	9
T-complex protein 1 subunit epsilon OS=Mus musculus GN=Cct5 PE=1 SV=1	5	11	6	11
Eukaryotic translation initiation factor 5A-1 OS=Mus musculus GN=Eif5a PE=1 SV=2	5	7	6	15
Tubulin alpha-4A chain OS=Mus musculus GN=Tuba4a PE=1 SV=1	35	51	28	48
Heterogeneous nuclear ribonucleoprotein A1 OS=Mus musculus GN=Hnrnpa1 PE=1 SV=2	11	8	6	12
14-3-3 protein beta/alpha OS=Mus musculus GN=Ywhab PE=1 SV=3	16	11	11	20
Cathepsin B OS=Mus musculus GN=Ctsb PE=1 SV=2	5	7	8	11
Chloride intracellular channel protein 1 OS=Mus musculus GN=Clic1 PE=1 SV=3	11	6	5	9
Probable ATP-dependent RNA helicase DDX5 OS=Mus musculus GN=Ddx5 PE=1 SV=2	7	6	5	13
1-phosphatidylinositol 4,5-bisphosphate phosphodiesterase gamma-2 OS=Mus musculus GN=Plcg2 PE=1 SV=1	5	10	7	9
60S ribosomal protein L4 OS=Mus musculus GN=Rpl4 PE=1 SV=3	8	8	5	10
Keratin, type II cytoskeletal 1b OS=Mus musculus GN=Krt77 PE=1 SV=1	8	10	7	11
Src kinase-associated phosphoprotein 2 OS=Mus musculus GN=Skap2 PE=1 SV=2	8	6	8	8
Malate dehydrogenase, cytoplasmic OS=Mus musculus GN=Mdh1 PE=1 SV=3	6	10	5	8
Serine/threonine-protein kinase PAK 2 OS=Mus musculus GN=Pak2 PE=1 SV=1	7	6	6	10
60S ribosomal protein L7a OS=Mus musculus GN=Rpl7a PE=1 SV=2	8	8	7	6
Splicing factor, proline- and glutamine-rich OS=Mus musculus GN=Sfpq PE=1 SV=1	5	6	8	10
Tubulin beta-4B chain OS=Mus musculus GN=Tubb4b PE=1 SV=1	55	81	56	87
V-type proton ATPase subunit B, brain isoform OS=Mus musculus GN=Atp6v1b2 PE=1 SV=1	5	6	5	13
Peptidoglycan recognition protein 1 OS=Mus musculus GN=Pglyrp1 PE=2 SV=1	7	6	7	9
60S acidic ribosomal protein P1 OS=Mus musculus GN=Rplp1 PE=3 SV=1	5	8	8	8

40S ribosomal protein S8 OS=Mus musculus GN=Rps8 PE=1 SV=2	8	6	5	9
Actin-related protein 2/3 complex subunit 3 OS=Mus musculus GN=Arpc3 PE=1 SV=3	6	6	9	6
Glia maturation factor gamma OS=Mus musculus GN=Gmfg PE=1 SV=1	5	7	8	7
60S ribosomal protein L13 OS=Mus musculus GN=Rpl13 PE=2 SV=3	7	7	6	7
14-3-3 protein eta OS=Mus musculus GN=Ywhah PE=1 SV=2	10	14	7	19
60S ribosomal protein L18 OS=Mus musculus GN=Rpl18 PE=2 SV=3	7	8	5	7
Macrophage-capping protein OS=Mus musculus GN=Capg PE=1 SV=2	7	5	7	8
Histone H2A type 2-A OS=Mus musculus GN=Hist2h2aa1 PE=1 SV=3	35	36	66	64
Keratin, type II cytoskeletal 73 OS=Mus musculus GN=Krt73 PE=1 SV=1	13	13	7	17
EF-hand domain-containing protein D2 OS=Mus musculus GN=Efhd2 PE=1 SV=1	6	5	9	6
Non-specific lipid-transfer protein OS=Mus musculus GN=Scp2 PE=1 SV=3	6	8	5	7
Non-POU domain-containing octamer-binding protein OS=Mus musculus GN=Nono PE=1 SV=3	5	5	6	9
40S ribosomal protein S19 OS=Mus musculus GN=Rps19 PE=1 SV=3	8	5	6	6
Proteasome subunit beta type-8 OS=Mus musculus GN=Psmb8 PE=1 SV=2	7	7	5	6
14-3-3 protein theta OS=Mus musculus GN=Ywhaq PE=1 SV=1	12	10	7	20
60S ribosomal protein L19 OS=Mus musculus GN=Rpl19 PE=1 SV=1	5	6	6	7
SH3 domain-binding glutamic acid-rich-like protein 3 OS=Mus musculus GN=Sh3bgrl3 PE=1 SV=1	7	7	2	7
Histone H2A.V OS=Mus musculus GN=H2afv PE=1 SV=3	14	8	9	19
Keratin, type II cytoskeletal 1 OS=Mus musculus GN=Krt1 PE=1 SV=4	10	11	7	11
Chitinase-like protein 4 OS=Mus musculus GN=Chil4 PE=1 SV=2	24	30	18	23
Histone H2AX OS=Mus musculus GN=H2afx PE=1 SV=2	21	13	12	17

Online Table S1- List of peptides present in all 4 groups of invasive protrusions and has minimum 5 detected peptides by mass spectrometry.

Title (or Source)	GO : ID	logP Value				WT 15min_GeneSet	Rap1b 15min_GeneSet	WT 60min_GeneSet	Rap1b 60min_GeneSet
		WT 15min	Rap1b 15min	WT 60min	Rap1b 60min				
podosome	GO:0002102	10	10	10	10	ACTR3 DBNL FERMT3 GSN HNRNPK LCP1 WDR1	ACTR3 DBNL FERMT3 GSN HNRNPK LCP1 WDR1	ACTR3 DBNL FERMT3 GSN HNRNPK LCP1 WDR1	ACTR3 DBNL FERMT3 GSN HNRNPK LCP1 WDR1
Arp2/3 protein complex	GO:0005885	10	10	10	10	ACTR2 ACTR3 ARPC1B ARPC2 ARPC3 ARPC4 ARPC5	ACTR2 ACTR3 ARPC1B ARPC2 ARPC3 ARPC4 ARPC5	ACTR2 ACTR3 ARPC1B ARPC3 ARPC4 ARPC5	ACTR2 ACTR3 ARPC1B ARPC2 ARPC3 ARPC4 ARPC5
actin filament	GO:0005884	10	10	10	10	ACTG1 ACTN1 ACTR3 CAPZB CORO1A EZR FLNAIQGAP1 LCP1 RAC2 VCL WDR1	ACTG1 ACTN1 ACTR3 CAPZB CORO1A FLNA IQGAP1LCP1 MYO1F RAC2 VCL WDR1	ACTG1 ACTN1 ACTR3 CAPZB CORO1A FLNA IQGAP1LCP1 RAC2 RCSD1 VCL WDR1	ACTG1 ACTN1 ACTR3 CAPZB CORO1A EZR FLNAIQGAP1 LCP1 MYO1F PTGES3 RAC2 RCSD1 VCL WDR1
actin filament bundle	GO:0032432	10	5	10	10	ACTN1 ACTN4 ANXA2 CNN2 FLNA LCP1 MYH9MYL12B PXN VCL ZYX	ACTN1 ACTN4 ANXA2 CNN2 FLNA LCP1 MYL12B VCL	ACTN1 ACTN4 ANXA2 CNN2 FLNA LCP1 MYH9MYL12B VCL ZYX	ACTN1 ACTN4 ANXA2 CNN2 FLNA LCP1 MYH9MYL12B VCL ZYX
stress fiber	GO:0001725	10	4	10	5	ACTN1 ACTN4 ANXA2 CNN2 LCP1 MYH9 MYL12B PXNVCL ZYX	ACTN1 ACTN4 ANXA2 CNN2 LCP1 MYL12B VCL	ACTN1 ACTN4 ANXA2 CNN2 LCP1 MYH9 MYL12B VCLZYX	ACTN1 ACTN4 ANXA2 CNN2 LCP1 MYH9 MYL12B VCLZYX
cluster of actin-based cell projections	GO:0098862	10	5	10	10	ACTN1 ACTN4 ACTR3 CAPZA1 CAPZA2 CAPZB CLIC1EZR FLNA HSP90AA1 HSP90AB1 MYH9 MYL12B MYL6VCL	ACTN1 ACTN4 ACTR3 CAPZA1 CAPZB CLIC1 FLNAHSP90AB1 MYL12B MYL6 TWF2 VCL	ACTN1 ACTN4 ACTR3 CAPZA1 CAPZB CLIC1 FLNAMYH9 MYL12B MYL6 TWF2 VCL	ACTN1 ACTN4 ACTR3 CAPZA1 CAPZA2 CAPZB CLIC1EZR FLNA HSP90AA1 HSP90AB1 MYH9 MYL12B MYL6TWF2 VCL
contractile actin filament bundle	GO:0097517	10	4	10	5	ACTN1 ACTN4 ANXA2 CNN2 LCP1 MYH9 MYL12B PXNVCL ZYX	ACTN1 ACTN4 ANXA2 CNN2 LCP1 MYL12B VCL	ACTN1 ACTN4 ANXA2 CNN2 LCP1 MYH9 MYL12B VCLZYX	ACTN1 ACTN4 ANXA2 CNN2 LCP1 MYH9 MYL12B VCLZYX
chaperonin-containing T-complex	GO:0005832		10		10		CCT2 CCT3 CCT5 CCT6A CCT7		CCT2 CCT3 CCT4 CCT5 CCT6A CCT7 CCT8 TCP1
contractile fiber	GO:0043292	4	4	5	4	ACTG1 ACTN1 ACTN4 ALDOA ANXA5 CALM1 CAPZBENO1 MYL12B RPL4 SPTAN1 SRI VCL	ACTG1 ACTN1 ACTN4 ALDOA ANXA5 CALM1 CAPZBENO1 MYL12B RPL4 RPL6 TWF2 VCL	ACTG1 ACTN1 ACTN4 ALDOA ANXA5 CALM1 CAPZBENO1 MYL12B RPL4 TWF2 VCL	ACTG1 ACTN1 ACTN4 ALDOA ANXA5 CALM1 CAPZBENO1 MYL12B RPL4 RPL6 SPTAN1 SRI TWF2 VCL
tubulin complex	GO:0045298	5	5		4	TPT1 TUBB TUBB4B	TPT1 TUBB TUBB4B		TPT1 TUBB TUBB4B
small ribosomal subunit	GO:0015935	5	10	5	10	NPM1 RPS17 RPS19 RPS2 RPS3 RPS3A RPS8 RPSA	DDX3X NPM1 RPS10 RPS14 RPS17 RPS18 RPS19 RPS2RPS3 RPS4X RPS7 RPS8 RPSA	DDX3X NPM1 RPS19 RPS3 RPS7 RPS8 RPSA	DDX3X NPM1 RPS14 RPS17 RPS18 RPS19 RPS2 RPS23RPS3 RPS3A RPS4X RPS6 RPS7 RPS8 RPS9 RPSA
secretory vesicle	GO:0099503	10	5	4	10	ACTN1 ACTN4 ALB ALDOA ANXA11 ANXA3 ANXA5CALR CAMP CAPZB CDC42 CKAP4 CTSG ELANE HSPA8HSPD1 ITGB2 LTF LYZ MPO MSN NCF2 PDIA3 PEBP1RAB8A SRI VDAC1	ACTN1 ACTN4 ALB ALDOA ANXA11 ANXA3 ANXA5CAMP CAPZB CCT6A CDC42 CTSG ELANE HSPA8HSPD1 ITGB2 LTF LYZ MPO PDIA3 PEBP1 VDAC1	ACTN1 ACTN4 ALB ALDOA ANXA3 ANXA5 CAMPCAPZB CDC42 CTSG ELANE HSPA8 HSPD1 ITGB2 LTFLYZ MPO PDIA3	ACTN1 ACTN4 ALB ALDOA ANXA11 ANXA3 ANXA5CALR CAMP CAPZB CCT6A CDC42 CKAP4 CTSG ELANEHSPA8 HSPD1 ITGB2 LTF LYZ MPO MSN NCF2 PDIA3PEBP1 SOD1 SRI TCP1 VDAC1

Online Table S2- GO Analysis of cellular component using Toppcluster tool, showing proteins as detected by mass spectrometry above 5 peptide threshold, in respective GO categories.

1	Absent in Rap1b 15min Fraction but present in WT 15min	Arsb Ap2b1 Sh3kbp1 Serpinb10 Kctd12 Cebpe Wasf2 Tmod3 Dnmt1 Hyou1 Cad Smarca5 Ctsc Urod Ube2n Srp68 Hdgf Lmnb2 Stom Cct4 Dhx15 Dctn2 Atp6v1g1 Eif3b Mapre2 Gatad2b Ruvbl2 Lbr Sept6 Sh3bp1 Uggt1 Glyr1 Acap2 Slc9a3r1 Prmt1 Sf3a2 Psmb2 G3bp1 Fam175b Vps35 Anp32a Ethe1 Rbmxl1 Hnrnpul2 Etfa Rpl35a Nsfl1c Gdi1 Coro1b Stip1 Eif4h Uba2 Alox15 Rplp2 Rab8b Msn Hspa9 Rab8a Ezr Krt14 Smad2 Calr Tmpo Tuba1c

	ID	Name	Source	pValue	FDR B&H	FDR B&Y	Bonferroni	Genes from Input	Genes in Annotation
1	1268678	Translation	BioSystems: REACTOME	2.628E-13	2.570E-10	1.918E-9	2.570E-10	16	165
2	1268686	GTP hydrolysis and joining of the 60S ribosomal subunit	BioSystems: REACTOME	1.148E-11	5.616E-9	4.191E-8	1.123E-8	13	119
3	1268679	Eukaryotic Translation Initiation	BioSystems: REACTOME	2.653E-11	6.486E-9	4.840E-8	2.594E-8	13	127
4	1268680	Cap-dependent Translation Initiation	BioSystems: REACTOME	2.653E-11	6.486E-9	4.840E-8	2.594E-8	13	127
5	1268681	Formation of a pool of free 40S subunits	BioSystems: REACTOME	5.461E-11	1.068E-8	7.972E-8	5.341E-8	12	107

2	Absent in WT 15min Fraction but present in Rap1b 15min	Anxa7 Caprin1 Rpl36a Elavl1 Fkbp1a Rars Sf3b3 Aip Crlf3 Spi1 Sf3b4 Cyfip2 Eif3j2 Cmtr1 Dhx9 Ssbp1 Rpl7 Phb Ppid Fmnl1 Esd Ceacam1 Hmha1 Glod4 Rps11 Cs Pfkp Ssu72 Eif3e Prep Actr1a Dpp3 Erh Fadd Mcm5 Vps25 Fhl3 Hars Ugp2 Dld Fis1 Gnb1

	ID	Name	Source	pValue	FDR B&H	FDR B&Y	Bonferroni	Genes from Input	Genes in Annotation
1	1269251	Nucleotide-binding domain, leucine rich repeat containing receptor (NLR) signaling pathways	BioSystems: REACTOME	8.690E-5	5.475E-3	2.589E-2	5.475E-3	2	48
2	1269818	Clearance of Nuclear Envelope Membranes from Chromatin	BioSystems: REACTOME	2.247E-3	3.284E-2	1.553E-1	1.416E-1	1	7
3	1269230	IRAK1 recruits IKK complex upon TLR7/8 or 9 stimulation	BioSystems: REACTOME	3.530E-3	3.284E-2	1.553E-1	2.224E-1	1	11
4	1269208	IRAK1 recruits IKK complex	BioSystems: REACTOME	3.530E-3	3.284E-2	1.553E-1	2.224E-1	1	11
5	1269254	The NLRP3 inflammasome	BioSystems: REACTOME	3.850E-3	3.284E-2	1.553E-1	2.426E-1	1	12

3	Absent in WT 60 min Fraction but preset in Rap1b 60min	Pstpip1 Fam107b Eif6 Aldh16a1 Raly Eif2s3x Prpf19 Rpl34 Ddx19a Coro1b Ssu72 Prmt1 Sf3a2 Eif3e Psmb2 G3bp1 Fam175b Prep Vps35 Arf2 Mat2a Olfm4 Vav1 Arrb2 Psmd12 Map4 Atp5c1 Acot7 Apex1 Psat1 Fkbp5 Rpl38 Idh2 Rpl15 Ppa1 Nsfl1c Rpl13a Aldh9a1 Tmed10 Snrnp200 Gsto1 Crlf3 Spi1 Sf3b4 Cyfip2 Eif3j2 Rab8b Cmtr1 Urod Ube2n Dhx9 Srp68 Hdgf Ssbp1 Stom Mcm6 Sqrdl Kcnab2 Gpd1l Me2 Mcm7 Bcat2 Eif5 Smarcc2 Glud1 Cast Lims1 Set Hk1 Rpn1 Rps24 Ganab Dcps C1qbp Psma1 Dld Alox5 Hnrnpul1 Snrpd3 Eif1 Etfb Nap1l1 Psmb3 Lbr Sept6 Sh3bp1 Uggt1 Cs Nme1 Sf3b3 Dnmt1 Hyou1 Cad Smarca5 Aip Ctsc Tomm22 Rcc1 Syk Fubp1 Eif3l Rpl31 Rpl30 Rpl24 Snrpg Ola1 Sept9 Dync1h1 Eif3f Ceacam1 Ap2b1 Sh3kbp1 Lmnb2 Rps4x Rpl22 Fh Rps6ka1 Mcm5 Rpn2 Ppib Casp3 Hnrnpul2 Dhx15 Park7 Tcp1 Gdi1

	ID	Name	Source	pValue	FDR B&H	FDR B&Y	Bonferroni	Genes from Input	Genes in Annotation
1	835393	superpathway of conversion of glucose to acetyl CoA and entry into the TCA cycle	BioSystems: BIOCYC	3.293E-5	9.276E-3	6.166E-2	1.422E-2	3	36
2	814926	Carbon metabolism	BioSystems: KEGG	4.294E-5	9.276E-3	6.166E-2	1.855E-2	4	114
3	MAP00020 Citrate cycle TCA cycle	MAP00020 Citrate cycle TCA cycle	GenMAPP	4.483E-4	3.990E-2	2.652E-1	1.937E-1	2	18
4	1270125	Citric acid cycle (TCA cycle)	BioSystems: REACTOME	5.005E-4	3.990E-2	2.652E-1	2.162E-1	2	19
5	PW:0000026	citrate cycle	Pathway Ontology	5.555E-4	3.990E-2	2.652E-1	2.400E-1	2	20

4	Absent in Rap1b 60min Fraction but Present in WT 60min	Plekho2 Edf1 Sugt1 Tpd52l2 Ube2v1 Tmpo Hnrnph1

	ID	Name	Source	pValue	FDR B&H	FDR B&Y	Bonferroni	Genes from Input	Genes in Annotation
1	83071	Tight junction	BioSystems: KEGG	1.332E-5	2.980E-3	1.987E-2	5.890E-3	6	170
2	1268677	Metabolism of proteins	BioSystems: REACTOME	1.348E-5	2.980E-3	1.987E-2	5.959E-3	16	1631
3	PW:0000159	Rab family mediated signaling	Pathway Ontology	3.395E-4	3.560E-2	2.374E-1	1.501E-1	2	9
4	1268688	L13a-mediated translational silencing of Ceruloplasmin expression	BioSystems: REACTOME	5.040E-4	3.560E-2	2.374E-1	2.228E-1	4	119
5	1268686	GTP hydrolysis and joining of the 60S ribosomal subunit	BioSystems: REACTOME	5.040E-4	3.560E-2	2.374E-1	2.228E-1	4	119

Online Table S3. List of uniquely expressed proteins. Only 1 peptide identified (Black) excluded from analysis, between 2-4peptied detected (Brown).5 or more peptide detected (Green). Gene from input identified by Go pathway analysis using ToppGene Suite application in the first column of the table below are underlined.

	Antibodies	Description	Catalogue	Company
1	Vinculin	Mouse Monoclonal Anti-Vinculin. Clone VIN-11-5	V4505	Sigma-Aldrich
2	MPO	Rabbit Polyclonal Anti-Human Myeloperoxidase	A0398	Dako
3	Pkm2	Rabbit monoclonal PKM2 (D78A4) XP®	4053	Cell Signaling
4	Ldha	Mouse monoclonal LDH-A Antibody (E-9)	sc-137243	Santa Cruz
5	β-Actin-HRP	Anti-beta Actin antibody [AC-15] (HRP)	ab49900	Abcam
6	CD31 / PECAM-1	Alexa Fluor® 488 anti-mouse CD31, Clone 390	102413	BioLegend
7	Hexokinase1	Rabbit monoclonal Hexokinase I (C35C4)	2024	Cell Signaling
8	G6PD	Rabbit Polyclonal G6PD antibody	A300-404A	Bethyl Lab
9	CD44	Rat Anti-Mouse CD44 antibody, Clone IM7 (RUO)	550538	BD Biosciences
10	Arp2	Mouse monoclonal Arp2 Antibody (E-2)	sc-137250	Santa Cruz
11	Myosin 2b	Rabbit monoclonal Myosin IIb (D8H8) XP® Antibody	8824	Cell Signaling
12	Rap1b	Rap1B (36E1) Rabbit monoclonal Antibody	2326	Cell Signaling
13	ZO-1	ZO-1 Monoclonal Antibody (ZO1-1A12), Alexa Fluor 488	339188	Fisher Scientific
	**Chemicals**	**Description**	**Catalogue**	**Company**
1	pHrodo	Invitrogen pHrodo Red 10,000 MW Dextran	P35372	ThermoFisher
2	2NBDG	(2-(N-(7-Nitrobenz-2-oxa-1,3-diazol-4-yl)Amino)-2-Deoxyglucose	N13195	ThermoFisher
3	LPS	Lipopolysaccharides from Escherichia coli O26:B6	L2654	Sigma-Aldrich
4	fMLP	N-Formyl-Met-Leu-Phe	F3506	Sigma-Aldrich
5	Gelatin matrix	Gelatin From Pig Skin, Oregon Green™ 488 Conjugate	G13186	ThermoFisher
6	Fibrinogen	Fibrinogen from murine plasma	F3879	Sigma-Aldrich
7	Ldha-Inhibitor	Lactate Dehydrogenase A Inhibitor, FX11	427218	Calbiochem
8	Akt-Inhibitor	MK-2206 2HCL	S1078	Selleck Chemicals
10	MCT-Inhibitor	α-Cyano-4-hydroxycinnamic acid	C8982	Sigma-Aldrich
11	Glycolysis Inhibitor	2-Deoxy-D-glucose	D8375	Sigma-Aldrich
12	Dextran	Dextran, Tetramethylrhodamine, 70,000 MW, Neutral	D1819	Life Technologics
13	Bend.3 Media	DMEM ATCC	30-2002	ATCC
14	Mounting Media	SlowFade Diamond Antifade Mountant	S36963	Molecular probes
15	Actin philloidin	Rhodamine phalloidin	R415	Life Technologics
16	LdhaI-II	Lactate Dehydrogenase Inhibitor II, GSK2837808A	533660	Millipore
17	Akt-Activator	Akt Activator II, SC79 - CAS 305834-79-1	123871	Millipore
18	Echinomycin	Isolated from Streptomyces echinatus (DSM 40013)	ALX-380-201-M001	Enzo
19	Histopaque	Histopaque®-1119 and Histopaque®-1077	11191/ 10771	Sigma
20	Cell Lysis buffer	RIPA Lysis and Extraction Buffer	89900	TermoFisher
	**Kits / Supplies**	**Description**	**Catalogue**	**Company**
1	Total Protein	Pierce BCA Protein Assay Kit	23227	Fisher Scientific
2	Albumin assay kit	BCG Albumin Assay Kit	MAK124	Sigma-Aldrich
3	Substrate for WB	SuperSignal™ West Pico PLUS Chemiluminescent Substrate	34580	ThermoFisher
4	Transwell 1.0um	Falcon® Permeable Support for 6 Well Plate with 1.0µm Transparent PET Membrane, Sterile, 1/Pack, 48/Case	353102	Corning
5	Transwell 3.0uM	24 mm Transwell with 3.0 µm pore polycarbonate membrane insert, TC-treated, w/lid, sterile, 24/cs	CLS3414	Sigma-Aldrich
6	Lactate assay kit	Lactate Dehydrogenase A/LDHA Assay Kit (Colorimetric)	KA0786	Novus Biologicals
7	ATP assay kit	ATP Determination Kit	A22066	TermoFisher

Online Table S4- List of antibodies, chemicals and kits.

References

1. Kawa S, Kimura S, Hakomori S, Igarashi Y. Inhibition of chemotactic motility and trans-endothelial migration of human neutrophils by sphingosine 1-phosphate. *FEBS Lett*. 1997;420(2-3):196-200.
2. Nesvizhskii AI, Keller A, Kolker E, Aebersold R. A statistical model for identifying proteins by tandem mass spectrometry. *Anal Chem*. 2003;75(17):4646-4658.
